# Advances in Perovskites for Photovoltaic Applications in Space

**DOI:** 10.1021/acsenergylett.2c01099

**Published:** 2022-07-09

**Authors:** Valentino Romano, Antonio Agresti, Rosaria Verduci, Giovanna D’Angelo

**Affiliations:** † Department of Physics, Politecnico di Milano, Piazza Leonardo da Vinci 32, 20133 Milano, Italy; ‡ Department of ChiBioFarAm, University of Messina, 98166 Messina, Italy; § CHOSE (Center for Hibrid and Organic Solar Energy), Department of Electronics Engineering, University of Rome Tor Vergata, 00133 Roma, Italy; ∥ Department of Mathematical and Computer Sciences, Physical Sciences and Earth Sciences, University of Messina, 98166 Messina, Italy; ⊥ CNR, Institute for Chemical-Physical Processes (IPCF), 98158 Messina, Italy

## Abstract

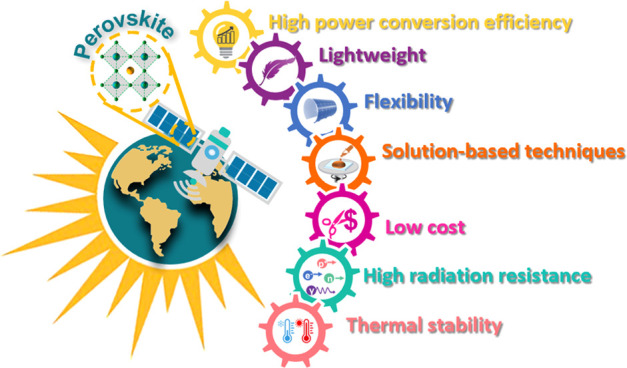

Perovskites have emerged as promising light harvesters in photovoltaics. The resulting solar cells (i) are thin and lightweight, (ii) can be produced through solution processes, (iii) mainly use low-cost raw materials, and (iv) can be flexible. These features make perovskite solar cells intriguing as space technologies; however, the extra-terrestrial environment can easily cause the premature failure of devices. In particular, the presence of high-energy radiation is the most dangerous factor that can damage space technologies. This Review discusses the status and perspectives of perovskite photovoltaics in space applications. The main factors used to describe the space environment are introduced, and the results concerning the radiation hardness of perovskites toward protons, electrons, neutrons, and γ-rays are presented. Emphasis is given to the physicochemical processes underlying radiation damage in such materials. Finally, the potential use of perovskite solar cells in extra-terrestrial conditions is discussed by considering the effects of the space environment on the choice of the architecture and components of the devices.

Since 1957, when the first “space-ship” (the soviet satellite Sputnik 1) was sent in Earth’s orbit, mankind’s curiosity has wondered what mysteries of the universe could be revealed. The following decades were characterized by an intense rush toward building the most advanced systems for space exploration. These efforts culminated in the moon mission by Apollo 11 in 1969. Afterward, thousands of spacecraft were sent into space with diverse purposes: studying phenomena on Earth (weather dynamics, tectonic movements, *etc*.), exploring our solar system (from the Sun to Pluto), and observing the universe surrounding us (galaxies, stars, and exoplanets). Furthermore, “orbiting laboratories” were built to conduct experiments at very peculiar conditions, such as in microgravity, *etc*., which are of interest for countless research fields, ranging from biophysics to agriculture, *etc*. The International Space Station is the biggest (and probably the most famous) example of such “orbiting laboratories”.

One of the main challenges ahead in the fabrication of spacecraft is their endurance because of the harsh conditions in which they operate. In fact, the atmosphere surrounding Earth acts as a shield against radiation and regulates the ambient temperature, so on-ground instruments work in a controlled environment. Quite differently, as altitude increases, the physicochemical properties of the environment change, making the working conditions of spacecraft orbiting Earth hostile. For these reasons, materials used in such spacecraft must show very high resistance not only to ensure their proper function but also because launching costs of space objects are expensive (ranging between ∼$30000 and ∼$1500 kg^–1^, depending on the vehicle’s characteristics),^1,2^ so the need for substitutions and maintenance must be minimized. Among all the components of such spacecraft, energy generation devices and electronic components play crucial roles because the former supply the energy needed to fuel the whole system, while the latter manage fundamental operations, such as ground communications.

In particular, modern spacecraft need several kilowatts of electric energy,^3,4^ which is usually produced through photovoltaic (PV) technologies because of the abundance of solar energy and safety requirements, making them preferable to alternatives such as batteries, fuel cells, and nuclear power.^5,6^ For example, the International Space Station contains four solar arrays made up by >260 000 Si-based solar cells (SCs) producing up to 120 kW.^4^ However, finding materials suitable for space applications is not a trivial task because there are several requirements that must be met: (i) resistance to the harsh space environment, (ii) low weight, (iii) high power conversion efficiency (PCE), and (iv) high gravimetric power (W kg^–1^).^5,7,8^ Moreover, the cost of PV technologies represents another important factor especially for the realization of extra-terrestrial habitable stations (for example, on the Moon and Mars) and for the new opportunities opened up by the privatization of the space industry (such as space tourism).^9^ Currently, the main materials used as light harvesters in SCs for space applications are Si and multijunctions based on III–V semiconductors. In particular, triple- and quadruple-junction SCs represent the best-performing devices available on the market from companies such as SolAero, Spectrolab, CESI, and Azur Space. One of the best-performing devices is the AlInGaP/AlInGaAs/InGaAs/Ge from Azur Space with an initial PCE = 31.8% and end of life PCE = 20.1% (at AM0 illumination, after irradiation with a dose of 10^16^ electrons cm^–2^ with 1 MeV energy).^10,11^ An example of a commercially available Si-based SC is another product of Azur Space which shows an initial PCE of 16.9% and an end of life PCE of 12.5% (under bombardment with a dose of 10^15^ electrons cm^–2^ with 1 MeV energy).^10^ However, these devices are rigid and thick (>100 μm, making them heavy with a gravimetric power of 0.4 W g^–1^ for InGaP/GaAs/Ge and 0.38 W g^–1^ for Si)^12^ and require complex and expensive fabrication processes in which scarce materials are used.^5,7,13−15^ Furthermore, several studies reported that multijunction SCs exhibit a performance degradation of ∼25% after receiving proton doses of 10^12^ particles cm^–2^,^16−18^ which can be accumulated in 3 years of exposure outside the Van Allen belts.^7^ Thus, there is an urgent need to find new materials that can provide useful alternatives to the space PV scenario. An interesting candidate is Cu(In,Ga)Se_2_ (CIGS), a lightweight (gravimetric power ∼3 W g^–1^)^12^ and radiation-resistant (showing only 10% decrease of PCE with incredibly high doses of 10^17^ electrons cm^–2^ with 1 MeV energy)^19,20^ sunlight absorber that can be exploited for the realization of flexible devices through low-cost processes.^21^ During the past decade, metal halide perovskites (MHPs) have attracted the interest of the PV terrestrial community because of their physicochemical properties that allow the realization of perovskite solar cells (PSCs) with PCEs exceeding 25%,^22^ rivalling the performances of much older technologies such as Si, CIGS, and CdTe. Figure 1 reports the crystalline structure of perovskites, whose general chemical formula is ABX_3_ (where A and B are cations and X is an anion).^23^

**Figure 1:**
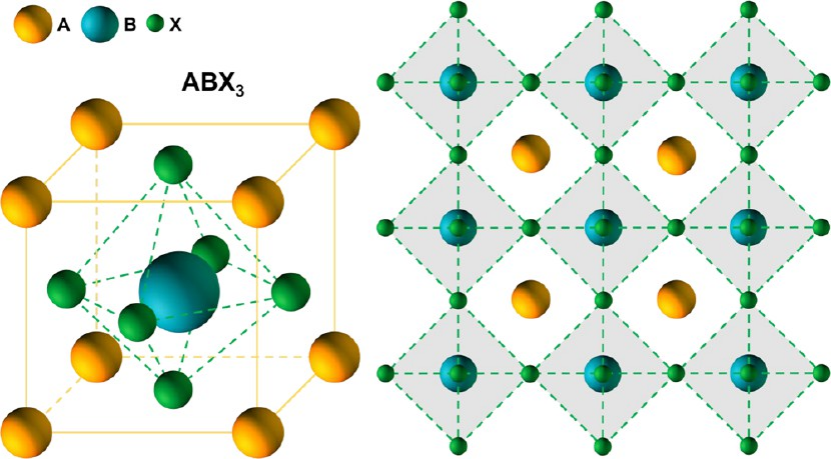
Schematic illustration of the perovskite crystal structure where A and B are cations and X is an anion.

For the case of MHPs, B is a divalent metal (for example, Pb^2+^, Sn^2+^, and Ge^2+^), X is a halide (such as I^–^, Cl^–^, and Br^–^), and A is a monovalent cation.^23^ The dimensions of A must fit within the voids formed by the (BX_6_)^4–^ octahedra, and it can be an elemental cation, such as Cs^+^, or a molecular cation, the most used being methylammonium (MA, CH_3_NH_3_^+^) and formamidinium (FA, H_2_NCHNH_2_^+^).^23^ The success of MHPs as light harvesters is due to several features which are optimal for PV technologies: high and panchromatic absorption coefficient through the visible region of the electromagnetic spectrum (>10^5^ cm^–1^), low exciton binding energies, low Urbach energies (tens of meV), long diffusion lengths of both electrons and holes (μm range), and high tolerance to defects.^23,25−30^ Moreover, the optoelectronic properties of MHPs, such as band gap energy, can be tuned by the proper engineering of their chemical composition,^30−33^ paving the way for their use in multijunction PV systems. Finally, PSCs present several other advantages such as low weight (due to their thin thickness <5 μm, with a gravimetric power of 23 W g^–1^),^12,34^ low fabrication cost through solution-processed techniques,^30,34^ and the possibility to realize flexible SCs (with a current PCE record >20%).^35^ In Figure 2 we present a detailed comparison of the main performance parameters between all the technologies discussed so far.

**Figure 2:**
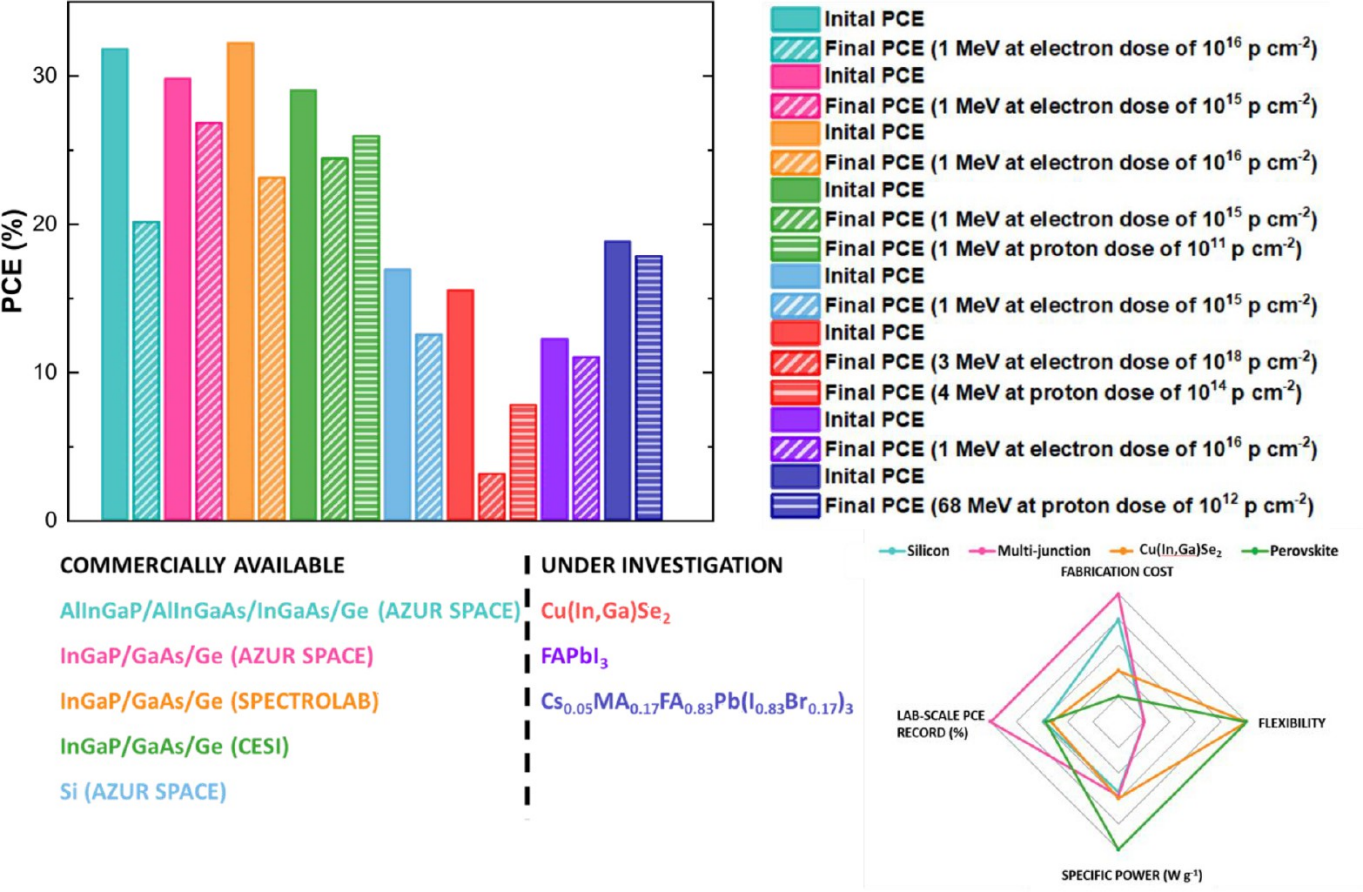
Comparison between several commercially available SCs used for space application and some representative devices of promising alternatives under investigation.

The aforementioned features of PSCs make them promising candidates for space PVs for many reasons. In particular, low weight and flexibility are pivotal requirements for space applications, not only to reduce the launching costs of spacecraft but also to allow the fabrication of roll-out solar arrays,^3,36,37^ which are currently produced by using rigid, thick, and heavy SCs (such as Si- and InGaP/GaAs/Ge-based devices). Furthermore, MHPs can be used as light harvesters in both single-junction as well as multijunction SCs with Si, CIGS, and Cu_2_ZnSn(S,Se)_4_.^38,39^ In this context, it is worth mentioning that MHPs/CIGS multijunction SCs are of particular interest for space applications because CIGS exhibits a high radiation resistance^19,20^ and can be produced in thin, flexible films.^40^ However, the moderate performance of CIGS-based SCs limits the practical use of this material. Conversely, MHPs/CIGS SCs have the potential for high efficiency, low weight (with a gravimetric power of 4 W g^–1^),^41^ and flexibility, which are fundamental for the realization of roll-out solar arrays. Thus, assessing the stability of PSCs while working in the space environment is fundamental to establishing their potential as a disruptive technology in the space sector.^42−44^

## Space Environment

Describing the space environment is not a trivial task because many phenomena occur, and they often correlate and influence each other. Herein, the discussion begins by considering the main source of space weather effects that our planet experiences: the sun. The solar outer atmosphere, the corona, continuously releases protons, electrons, helium nuclei, and a small amount of heavier ions.^45^ The temperature of these ejected particles (which constitute the so-called solar wind) is sufficiently high to form a *plasma*, one of the seven factors that the NASA Marshall Space Flight Center has identified for the description of the space environment.^46^ Such plasma spreads into space at 300–900 km s^–1^ with a very low density (∼30 particles cm^–3^) and a temperature of ∼105 K.^45^

**Figure 3:**
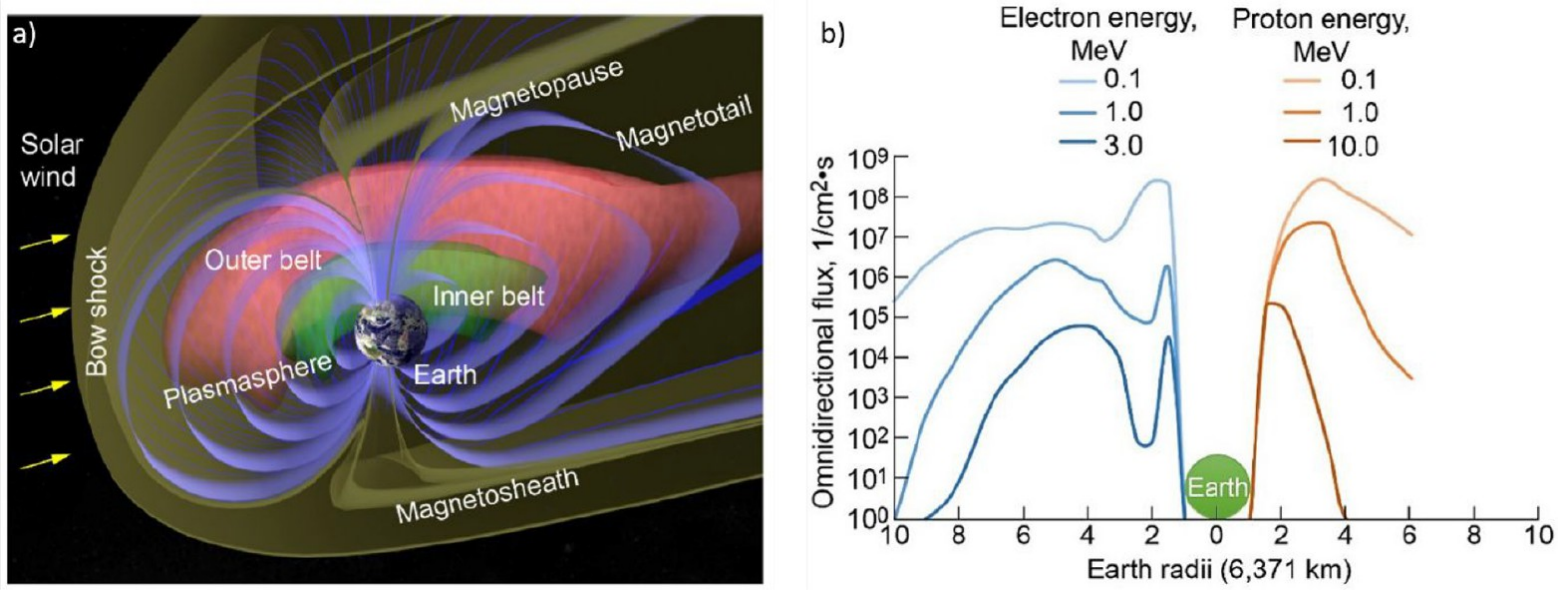
(a) Representation of the main components of earth’s atmosphere. The green and pink pseudotoroids indicate the inner and outer Van Allen radiation belts, respectively. (b) Flux of electron and proton radiation as a function of altitude (expressed in Earth radii). Reprinted with permission from ref (47). Copyright 2020 NASA.

When the solar wind approaches Earth, interactions with the *geomagnetic field* occur. The geomagnetic field, which arises from the motion of iron atoms in the Earth’s liquid core, is another factor affecting the space environment because it is responsible for a plethora of fundamental phenomena.^45,46,48,49^Figure 3a summarizes the main components of the so-called magnetosphere. The incoming plasma has a speed which is greater than that of Alfven waves (*i.e.*, transverse magnetohydrodynamic waves)^48^ in the solar wind medium; thus, when the plasma hits the Earth’s magnetosphere, a shock wave (and consequently a bow shock) occurs (this magnetohydrodynamic process is the equivalent observed in our atmosphere when objects travel at supersonic speeds).^45,48,49^ It was estimated that only a small fraction (>1%) of the incoming solar wind can cross the bow shock.^45^ The region behind this impact zone (termed the magnetosheath) is characterized by turbulent wave motion and the presence of plasma hotter than that arriving from the sun (because of the strong deceleration experienced, which reduces the speed of the solar wind to ∼50 km s^–1^, causing the dissipation of such energy into heat).^45,48,49^ The remaining plasma flows on the geomagnetic field lines, stretching the magnetosphere in the antisunward side and forming a magnetotail which extends into space for distances even beyond 100*R*_E_.^45,48,49^

The solar wind is not the only source of plasma that must be taken into account. It can derive from astronomical events occurring outside our solar system (*i.e.*, from cosmic rays),^47^ and it can be indirectly produced by high-energy photons (UV, X-rays, and γ-rays emitted by solar activity and cosmic rays) that can easily ionize atoms and molecules in the uppermost layers in the atmosphere.^49^ Thus, in general, the atmosphere can be regarded as made up by a neutral component (troposphere, stratosphere, and lower thermosphere) whose constituents are not ionized and an ionosphere (upper thermosphere, mesosphere, and lower exosphere) characterized by the presence of cold plasma (with energy in the eV range)^45^ forming a toroidal region of space termed the plasmasphere.^48,49^ The region that separates the plasmasphere from the solar wind is the magnetopause.^49^

It is fundamental to emphasize that some of the fastest charges can penetrate the outermost geomagnetic field lines and can eventually get trapped by inner field lines or reach Earth’s atmosphere.^47,49^ In the latter case, collisions between protons and nuclei with the atmosphere’s constituents trigger many decay pathways that lead to the emission of many subatomic particles, such as neutrons, muons, kaons, pions, etc.^47^ Conversely, particles that get trapped by geomagnetic field lines form two main regions called Van Allen radiation belts:^45,49^ the inner belt (situated in the region between 1.5 and 3.5 *R*_E_ from Earth’s center) and the outer belt (in the range of 3–7 *R*_E_).^47^ The first one captures high-energy protons, while the second one typically stops electrons; thus, spacecraft orbiting at such altitudes must be resistant to this kind of environment.^45,47,49^Figure 3b shows the flux (varying between 10^3^ and 10^8^ particles cm^–2^ s^–1^)^50−52^ and energy distribution of protons and electrons around Earth as functions of altitude (expressed in *R*_E_ units). Thus, a third factor affecting the space environment can be introduced: *radiation* (responsible for ∼40% of space-related issues) from both particles and photons.

The fourth factor is due to the *neutral atmosphere* that comprises three main effects: atmospheric drag, the presence of atomic oxygen, and high vacuum conditions. The former must be taken into account to optimize orbital shape and altitude; thus, it will not be further analyzed in this Review because it is related to orbital mechanics and aerospace engineering. Quite differently, atomic oxygen (which constitutes 80% of the upper atmosphere composition, *i.e.*, in the range of 200–800 km) can be detrimental to the operation of the spacecraft’s components.^53^ Although its density is not high, atomic oxygen can corrode surfaces it interacts with and can break the chemical bonds of many materials because of the high average impact energy (4–5 eV) resulting from collisions with spacecraft orbiting at a speed of 7–8 km s^–1^.^49,54^ Finally, the density of the atmosphere decreases with increasing altitude (ranging from 1 bar at sea level to 10^–11^ bar at ∼1000 km),^55^ leading to huge pressure gradients between the interior and the external part of the spacecraft.

The fifth factor described by the NASA Marshall Space Flight Center is the presence of *debris* (due to meteoroids and comets or human space activities), which is extremely dangerous because collisions with spacecraft can cause severe damage. A huge amount of space debris orbits around Earth, with dimensions and concentrations depending on the altitude^49,56^ (according to statistics from ESA, as of May 2022, more than 130 million pieces of debris were present in the space surrounding Earth).^57^ These objects represent a severe threat because a critical mass threshold exists that, if reached, can trigger avalanche collisions, eventually causing the destruction of several spacecraft.^57,58^ Thus, the presence of space junk must be reduced by proper actions exploiting one of the several existing strategies, such as recovery, deorbiting, and laser removal.^56,59,60^

*Thermal fluctuation* represents the sixth factor and is one of the main sources of damage in spacecraft (it accounts for 11%).^56^ It arises because of (i) solar radiation, (ii) albedo radiation (*i.e.*, solar radiation reflected back to space by Earth’s surface), (iii) thermal energy from other celestial bodies, (iv) space average temperature at 4K, and (v) thermal energy sources on board the spacecraft (such as electric energy dissipation, fuels, *etc*.).^49^ Furthermore, the orbital motion of spacecraft causes thermal cycling because of the alternation of light and dark hours.

Finally, the seventh factor accounts for *solar activity*, which concerns solar phenomena (such as solar flares and coronal mass ejections), characterized by the emission of huge quantities of high-energy particles and photons. Such astronomical events influence almost all the factors mentioned so far: both plasma and radiation levels are increased, the geomagnetic field experiences stronger interactions with the solar wind, and thermal fluctuations become more intense. Thus, solar activity has a very detrimental effect on spacecraft, but it is hardly ever predictable, making the space environment even more hostile.

## Main Effects of the Space Environment on Materials

The seven factors discussed in the previous section affect materials used for space applications in many ways. However, while some of them can be faced by proper engineering of spacecraft and shielding strategies, others have detrimental effects on the physicochemical properties of materials. Thus, it is essential to understand such degradation processes to advance the research of stable and performing materials.

For example, the presence of debris (around Earth, as well as objects encountered during space missions) is dangerous for the safety of the whole spacecraft; thus, it will not be further discussed here because it has no specific effects on the physicochemical properties of materials. Similarly, the effects of the neutral atmosphere can be faced by the proper engineering of the spacecraft. In particular, the interaction with atomic oxygen can lead to severe corrosion, and that can be mitigated through the use of anticorrosive materials and coatings.^61,62^ Furthermore, the vacuum is responsible for several detrimental phenomena, such as material outgassing and effusion, evaporation, adhesion, cold welding, sublimation, *etc*.^49,56^ Thus, pressure control and sealing strategies are fundamental to avoid these dangerous issues. Regarding the geomagnetic field, its degradation pathways are related to those from trapped charged particles; thus, its effects on materials are the same as those due to particle radiation. The plasma environment is another serious threat to the long-term function of spacecraft. In general, it can be classified into hot and cold plasma (the former is the one generated by solar activity, and the latter comes mainly from Earth’s atmosphere).^56^ It is worth emphasizing that cold plasma can turn into hot plasma because of bombardment by solar wind. Thus, both cold and hot plasma are dangerous, because they can induce surface charging and consequently electrostatic discharge, power loss, and short circuit in electronic and photovoltaic components.^49,56,63,64^ Fortunately, such effects can be mitigated through shielding strategies and active control of potential.^56,65,66^ Finally, the main effect arising from solar activity is a variation in the amount and properties of the irradiated plasma, *i.e.*, flux and energy of the emitted particles.

Thus, the main factors that affect the stability and physicochemical properties of materials used for energy generation devices and electronic components are thermal fluctuations and radiation. In particular, fluctuations of the temperature (which can be due to alternation between day and night hours, infrared radiation from Earth, albedo radiation, *etc*.) can cause thermal expansion and contraction, vibration, and eventually the rupture of some materials.^49,56^ Many strategies can be exploited to reduce such effects, both passive and active, such as the use of resistant materials and heat exchangers, as well as temperature compensation systems.^56,67^ However, thermal stability must be properly addressed especially with respect to phase transitions that can deeply modify the physicochemical properties of materials, making them unusable.

Radiation-induced degradation is probably the most studied and complicated factor that needs to be considered when dealing with the space environment. Because radiation damage depends on the type of interactions developed within materials, two distinct types of radiations can be distinguished: directly ionizing (*i.e.*, charged particles which strongly interact with the electron clouds) and indirectly ionizing (*i.e.*, neutrons and photons whose interaction with materials can cause nuclear transformations or liberate ionizing radiation, respectively).^68^ In general, the effects of radiation on materials are impurity production (because of neutron capture or the neutralization of charged radiation such as protons or α-particles), atom displacement (due to nuclei recoiling after scattering events), energy release, and ionization.^68^ In particular, α-particles and protons are dangerous sources of radiation damage as they cause the formation of defects within the material structure.^68^ However, the nuclear stopping power is proportional to *E*^–2^; thus, smart sealing strategies can efficiently act as shields for low-energy protons, α-particles, and neutrons.^68,69^ Quite differently, high-energy particles (in the range of tens of MeV) can penetrate within materials and cause several interactions before being completely stopped.

Thus, knowing the energy distribution and flux of radiation in the space environment is also fundamental to thoroughly assess their detrimental effects on materials’ stability. For example, in low Earth orbit (which is the region of altitudes between 160 and 2000 km) the radiation environment is typically dominated by electrons (with 1 MeV energy at a flux of 6 × 10^3^ cm^–2^ s^–1^) and protons (with 100 keV energy at a flux of 10^4^ cm^–2^ s^–1^).^70^ Moreover, the flux of both protons and electrons around Earth varies between 10^3^ and 10^8^ cm^–2^ s^–1^, depending on both altitude and solar activity.^45,49,51,52^ Thus, accelerated tests (which are conducted at much higher accumulated doses) are needed to ascertain the long-term stability of materials for space applications; for example, doses up to 10^12^ particles cm^–2^ of protons are often investigated which, for the case of 68 MeV energy, are typically accumulated in ∼50 years at the International Space Station orbit (altitude ∼400 km).^41^

It is worth mentioning that radiation-resistant technologies are of great interest also for on-ground applications. Indeed, there are many radioactively polluted zones on Earth, located near densely populated regions, where no economic activity will occur because new construction is forbidden.^71^ Examples of these areas are Chernobyl, Fukushima, and nuclear test sites such as those in Nevada (United States) and Semipalatinsk (Kazakhstan).^72^ The economic growth and rebirth of these regions will be possible only through access to energy sources, and radiation-resistant PV technologies can be the solution to this hurdle. Thus, research in this field can also have an important impact for life on Earth.

## Radiation Resistance of Metal Halide Perovskites

Assessing the resistance of MHPs to the space environment is not a trivial task because of the intrinsic instability that such materials experience under photoexcitation. Thus, distinguishing the effects of each source of degradation can be tricky. Nonetheless, several studies addressed the resistance of MHPs in the harsh space environment, with particular emphasis on radiation-induced effects. In the following sections, we will review and discuss the main results about the mechanisms responsible for performance losses in PSCs.

### Resistance to Protons and Electrons

**Figure 4:**
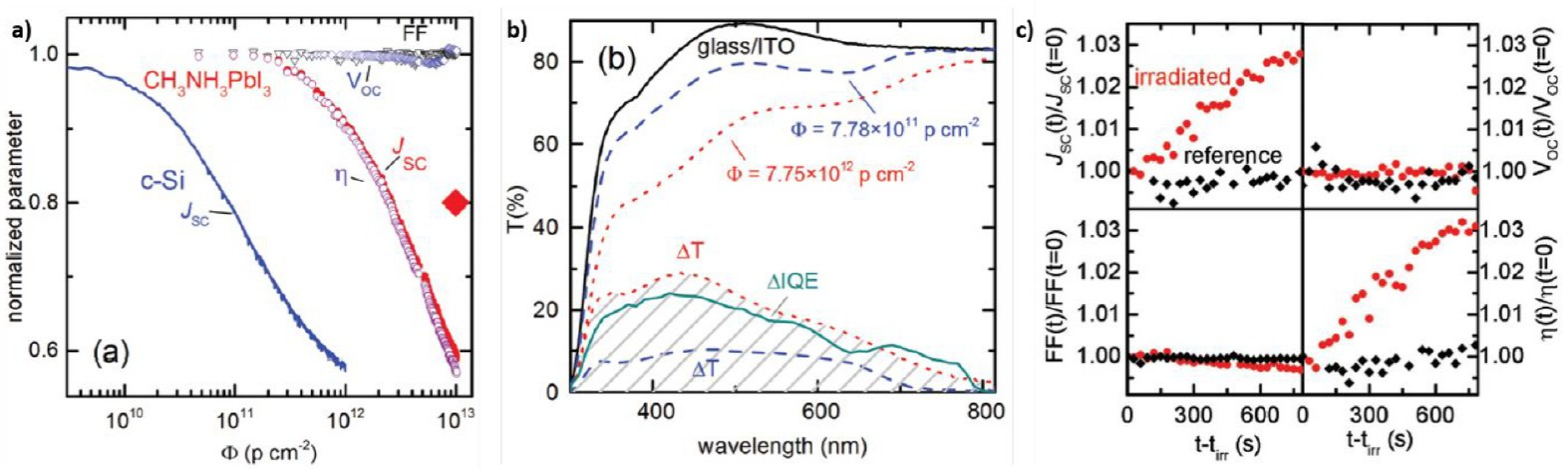
(a) Normalized *J*_SC_ (red dots), *V*_OC_ (blue rhombuses), FF (black triangles), and PCE (purple dots, termed η) for MAPI-based SCs reported as functions of the accumulated proton doses. For comparison, the *J*_SC_ of a Si photodiode is also shown (blue line). The red rhombus refers to the PCE obtained when the correction, due to the glass/ITO substrate losses, is taken into account. (b) Transmittance of the glass/ITO substrate at nonirradiated conditions (black line) and at proton doses of 7.78 × 10^11^ particles cm^–2^ (blue dashed line) and 7.75 × 10^12^ particles cm^–2^ (red dotted line). The proton-induced variations, with respect to nonirradiated case, are shown as Δ*T* with the same color legend. The difference in internal quantum efficiency (ΔIQE) is also reported (aqua green line). (c) Comparison of the normalized photovoltaic parameters for reference and irradiated devices, measured after the irradiation experiments. Reprinted with permission from ref (71). Copyright 2016 Wiley.

The starting point of this discussion concerns the effects of ionizing radiation on MHPs. In particular, Lang *et al*. demonstrated the proton hardness of MAPbI_3_ by investigating the photovoltaic performances of p-i-n solar cells.^71^ In particular, they irradiated their samples with protons having 68 MeV energy and monitored the performances of their devices up to 1.02 × 10^13^ particles cm^–2^ accumulated doses. Interestingly, the *V*_OC_ and FF remain almost constant in the whole range of investigated doses (Figure 4a), while *J*_SC_ (and consequently PCE) decreases for doses >2 × 10^11^ particles cm^–2^. However, it must be taken into account that performance losses occur also because of degradation from the other components of SCs. In particular, radiation affects the transmittance of the glass substrates because of the formation color centers within the glass structure.^73,74^ Thus, the authors analyzed their substrates and observed severe variations of their transmittance (Figure 4b). When such an effect is taken into account, only a 20% reduction of the JSC is observed at a dose of 10^13^ particles cm^–2^ (red rhombus in Figure 4a), making the radiation tolerance of such PSCs far higher than that of c-Si photodiodes (that suffer from degradation already at doses of ∼10^10^ particles cm^–2^, *i.e.*, 3 orders of magnitude lower than that of PSCs). Furthermore, the authors observed another interesting phenomenon after monitoring the *J*–*V* curves of their irradiated devices after the end of the irradiation test. They detected an increase in both *J*_SC_ and PCE (while *V*_OC_ and FF still remained the same, Figure 4c) even with respect to the reference nonirradiated devices, underlying the self-healing effect experienced by MHPs. The value of *J*_SC_ depends on many factors, including the presence of defects and imperfections in the crystal structure of the materials composing the device. Because proton irradiation is known for its detrimental effects due to the displacement of atoms from their lattice sites and nuclear reactions due to proton capture (just to name few), the authors propose that such observations are due to the formation of imperfections in the MHP structure that can be passivated once the disturbance is turned off. They suggest that proton irradiation causes the dissociation of C–H and N–H bonds with the consequent release of H^+^ ions within the MHP structure. When the irradiation ends, these H^+^ ions passivate the defects formed during the irradiation and those that were already present before the radiation treatment; thus, *J*_SC_ (and PCE) increases accordingly, and the final performance is also superior to that of the reference devices (Figure 4b). Although this seems a plausible explanation of the observed results, the authors do not provide any further experimental investigation of such phenomenon; thus, further investigations are required.

**Figure 5:**
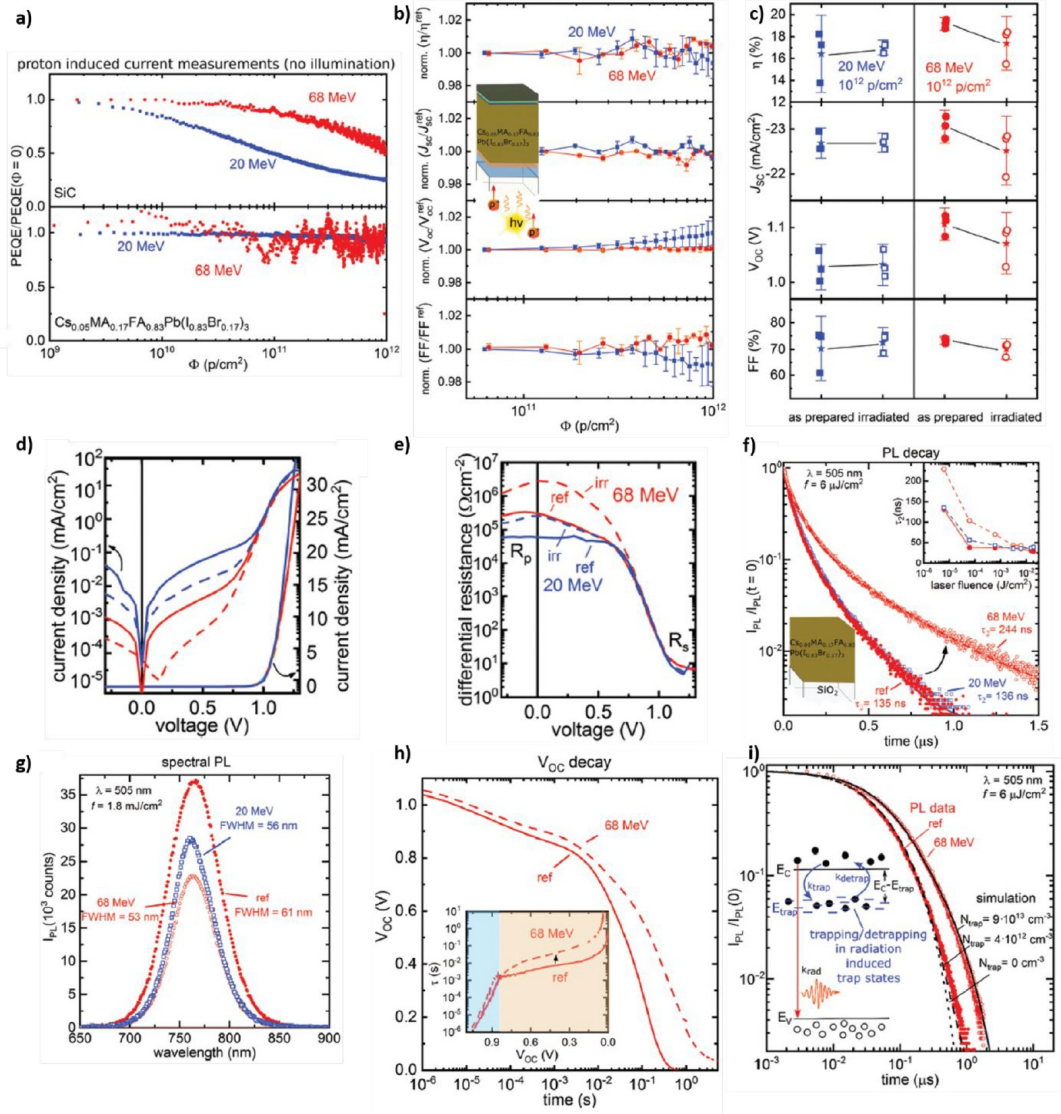
(a) Relative proton induced quantum efficiency (PEQE/PEQE(Φ = 0)) for SiC (top) and Cs_0.05_MA_0.17_FA_0.83_Pb(Br_0.17_I_0.83_)_3_ irradiated with protons with energies of 20 and 68 MeV. (b) Relative variation (with respect to measurements conducted under no bombardment) of the photovoltaic parameters *J*_SC_, *V*_OC_, FF, and PCE (η in the figure) for Cs_0.05_MA_0.17_FA_0.83_Pb(Br_0.17_I_0.83_)_3_-based PSCs under irradiation with protons with energies of 20 MeV (blue line) and 68 MeV (red line) and variable flux. (c) Comparisons of the photovoltaic parameters measured before and after irradiation of the devices with protons with energies of 20 (blue line) and 68 (red line) MeV. (d) Linear and semilogarithmic *J*–*V* curves measured in dark conditions for nonirradiated devices (solid lines) and irradiated with protons having energies of 20 MeV (blue line) and 68 MeV (red line). (e) Comparisons of the differential resistance for reference and bombarded devices. (f) Normalized PL decays of reference (full red circles) and irradiated [with protons having energies of 20 MeV (empty blue squares) and 68 MeV (empty red circles)] Cs_0.05_MA_0.17_FA_0.83_Pb(Br_0.17_I_0.83_)_3_ samples (deposited onto quartz). The inset reports values of τ_2_ calculated at different laser fluences. (g) Comparison of the PL spectra for reference and irradiated Cs_0.05_MA_0.17_FA_0.83_Pb(Br_0.17_I_0.83_)_3_ samples (deposited onto quartz) (the same legend of panel f applies to this case). (h) Measured VOC decay for reference (solid line) and irradiated (dashed line) devices. (i) Comparisons of simulated and measured PL decays of Cs_0.05_MA_0.17_FA_0.83_Pb(Br_0.17_I_0.83_)_3_ thin films (deposited onto quartz) before and after irradiation with protons having 68 MeV energy. Reprinted with permission from ref (7). Copyright 2019 Royal Society of Chemistry.

In a subsequent work, the same group analyzed an identical solar cell architecture using a triple-cation, mixed-halogen MHP (Cs_0.05_MA_0.17_FA_0.83_Pb(Br_0.17_I_0.83_)_3_), with the aim of understanding the radiation hardness of such kinds of light harvesters.^7^ Because the stopping power of particle radiation depends on the impact energy, the authors analyzed the effects of proton irradiation by using several values of the impact energy, namely, 10 ± 3, 20 ± 3, and 68 ± 1 MeV. These values represent well the space environment as higher energies are found rarely (typically only during intense solar activity).^50^ Protons with the lower energy (10 MeV) usually are completely stopped within the glass substrate (as their penetration depth is ∼1 mm). Quite differently, 20 and 68 MeV protons can penetrate within the perovskite layer and experience inelastic scattering (from the electron clouds, thus causing ionization or excitation of the constituents) or elastic scattering (from the nuclei, which can lead to the displacement of atoms, *i.e.*, the formation of vacancies and interstitial defects). As a first step toward understanding the radiation hardness of MHPs, the authors evaluated the variation of the proton-induced quantum efficiency (PEQE). This quantity arises from the fact that ionizing radiation causes the formation of electron–hole pairs and, consequently, leads to a current density within the irradiated material. In the investigated triple-cation perovskite, a *J* ≈ 290 nA cm^–2^ was induced by protons with 20 MeV energy and a flux (φ) equal to 149 pA cm^–2^. As a result, the PEQE (J/φ) is ∼2 × 10^3^; as a consequence, each proton leads to the generation of more than 2 × 10^3^ electron/hole pairs (some are inevitably lost because of recombination). Long-lasting irradiation usually disrupts the crystal lattice; thus, a first piece of interesting information can be obtained by monitoring how the PEQE varies with increasing accumulated dose. Figure 5a reports the comparison between the evolution of PEQE of triple-cation perovskite and a SiC diode (a material tolerant to high-energy proton irradiation),^75^ revealing that the PEQE for the investigated MHP experiences a small (7%) reduction at both 20 and 68 MeV proton irradiation (with an accumulated dose of 10^12^ particles cm^–2^). Quite differently, the PEQE of SiC decreases (considering an accumulated dose of 10^12^ particles cm^–2^) by 50% (at 20 MeV) and 75% (at 68 MeV). These results suggest that the crystal structure of MHPs is far more resistant to proton irradiation with respect to a benchmark material such as SiC, also paving the way for MHP-based proton detectors. The radiation resistance of such devices was also demonstrated by measuring the *J*–*V* curves during irradiation. Figure 5b reports the evolution of the photovoltaic parameters up to doses of 10^12^ particles cm^–2^, revealing negligible changes in their values for both 20 and 68 MeV energies. The same devices were measured again after 2 weeks from the irradiation experiment (as radiation levels had to decay to tolerable values), and the performance was compared to that obtained before proton irradiation (Figure 5c). The results clearly show that 20 MeV protons do not cause any significant variation of the photovoltaic performance, while the devices irradiated with 68 MeV protons show a decline of all the performance metrics, probably because of the increase of Schockley–Read–Hall (SRH) recombination. With the aim of deeply understanding the effects of proton-induced defects, the authors conducted several characterizations. First, they measured the dark *J*–*V* curves (Figure 5d) of the pristine and irradiated devices which revealed two main features: an improved rectification after irradiation and a shift (from 0 to ∼150 mV) of the *J*–*V* curve in the case of irradiation with 68 MeV protons. Furthermore, by computing the differential resistance (*R*_diff_ = Δ*V*/Δ*J*), they surprisingly observed an increase of the parallel (shunt) resistance (*R*_P_) as the accumulated dose increased (as shown in Figure 5e). Higher values of *R*_P_ imply lower bulk recombination of charge carriers (associated with SRH phenomena or to shunts in the polycrystalline film); thus, such results suggest that irradiation by high-energy protons would reduce the presence of defects within the MHP layer. To elucidate the inconsistency between this conclusion and the partial decline of the photovoltaic performance, the authors recorded PL and *V*_OC_ decays. In particular, Figure 5f reports PL transient spectra recorded on pristine and irradiated (with 20 and 68 MeV protons) perovskite samples (deposited on glass substrates). Surprisingly, the longest lifetime was measured for the sample irradiated with 68 MeV protons (τ_2_ = 244 ns), which is quite unexpected because higher lifetimes are associated with reduced recombination events. Thus, even this measurement would suggest that irradiation with 68 MeV protons induces a reduction of recombination pathways in MHPs. If this was true, then a higher PL intensity should be observed, but this is not the case as demonstrated by the reduced PL yield (Figure 5g), the lowest value being associated with the sample irradiated with the most energetic protons. Finally, another very interesting observation comes from *V*_OC_ decays before and after irradiation with 68 MeV protons (Figure 5h). Prior to irradiation, *V*_OC_ becomes 0 in ∼1s while the irradiated sample shows a slower decay and an additional decay step after 1 s. By considering the carrier density (*n*) as given by
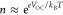
where *e* is the elementary charge, *k*_B_ Boltzmann’s constant, and *T* the absolute temperature, a recombination lifetime can be defined according to
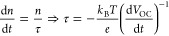
This quantity is reported in the inset of Figure 5h, revealing that τ (and so the decay of *V*_OC_) follows two regimes: (i) above 0.86 V, where both irradiated and pristine samples show the same exponential increase of τ with increasing *V*_OC_; (ii) below 0.86 V, in which irradiated devices are characterized by values of τ which are an order of magnitude higher with respect to those of the nonirradiated solar cells. The presented results point toward prolonged lifetimes caused by trapping–detrapping of charge carriers, a phenomenon that was discussed by Hornbeck and Haynes^76^ (and observed for other materials such as polycrystalline silicon).^77^ For this reason, by following the model by Hornbeck and Haynes, the authors simulated the PL decay of their nonirradiated and irradiated (with 68 MeV protons) MHP considering radiative, Auger recombination, and the trapping–detrapping process due to irradiation-induced traps. As clearly shown in Figure 5i, the PL decay of the nonirradiated sample is reproduced with a very good agreement by considering a trap density (*N*_trap_) equal to 0 cm^–3^ while by considering *N*_trap_ = 9 × 10^–13^ cm^–3^, the PL decay of the irradiated sample (68 MeV protons) is obtained. Thus, trapping–detrapping of the charge carrier is the source of the apparent increase of charge carrier lifetimes and *V*_OC_ behavior. The authors propose that such trap states are due to iodine interstitial defects because theoretical investigations have shown that such kinds of defects have a low formation energy^78^ and can trap both holes and electrons^79^ in MAPbI_3_.

The works discussed so far concern the stability of MHPs to high-energy particle radiation (*i.e.*, protons with energies ≥20 MeV). A study by Miyazawa *et al*.^70^ noted that this choice may cause misleading results about the radiation stability of MHPs because, according to their results, particles with such energies can penetrate the perovskite layer causing few collision events. In particular, they investigated the effects of electron and proton beam (EB and PB) irradiation on the photovoltaic performance of MA- and FA-based mixed halogen perovskites. Their results evidenced clearly the superior radiation tolerance of MHP solar cells to radiation-induced damage by 1 MeV EB, which retain ∼90% of the PCE at accumulated doses of 10^16^ particles cm^–2^. For comparison, benchmark light harvesters for space photovoltaics such as Si and InGaP/GaAs/Ge suffer from severe losses at such doses, with a PCE retention of ∼60% for both cases.^80,81^ Regarding irradiation with PB, the authors investigated through Stopping and Range of Ions in Matter/Transport of Ions in Matter (SRIM/TRIM) simulations the depth of penetration of protons with energies ranging from 50 keV to 60 MeV. According to their results, PB with 50 keV energy have a penetration depth corresponding to the position of the perovskite layer, while PB with higher energies (especially those in the range of tens of MeV) can cross the perovskite layer causing few collision events. For this reason, irradiation of perovskite solar cells with PB having 50 keV of energy at doses up to 10^15^ particles cm^–2^ was performed, revealing again the high radiation hardness of MHPs, which could retain ∼50% of PCE (devices produced with Si and InGaP/GaAs/Ge could not survive such tests).^82,83^

It should be noted that the work from Miyazawa *et al*. emphasizes that PB with high MeV energy can cross the perovskite layer causing little damage. For this reason, the radiation hardness of MHPs should be assessed by using PB that can effectively interact with the perovskite layer of SCs, which is made possible by tuning the energy of the investigated radiation probe. However, the works from Lang *et al*. and Brus *et al*. clearly show that PSCs suffer from performance losses also when MeV energies are used. This outcome may be due to losses associated with the other layers of the PSCs, but according to the presented results, this may not be the actual case. It is then clear that further and more thorough investigations are needed to completely assess the radiation stability of MHPs under radiation exposure.

**Figure 6:**
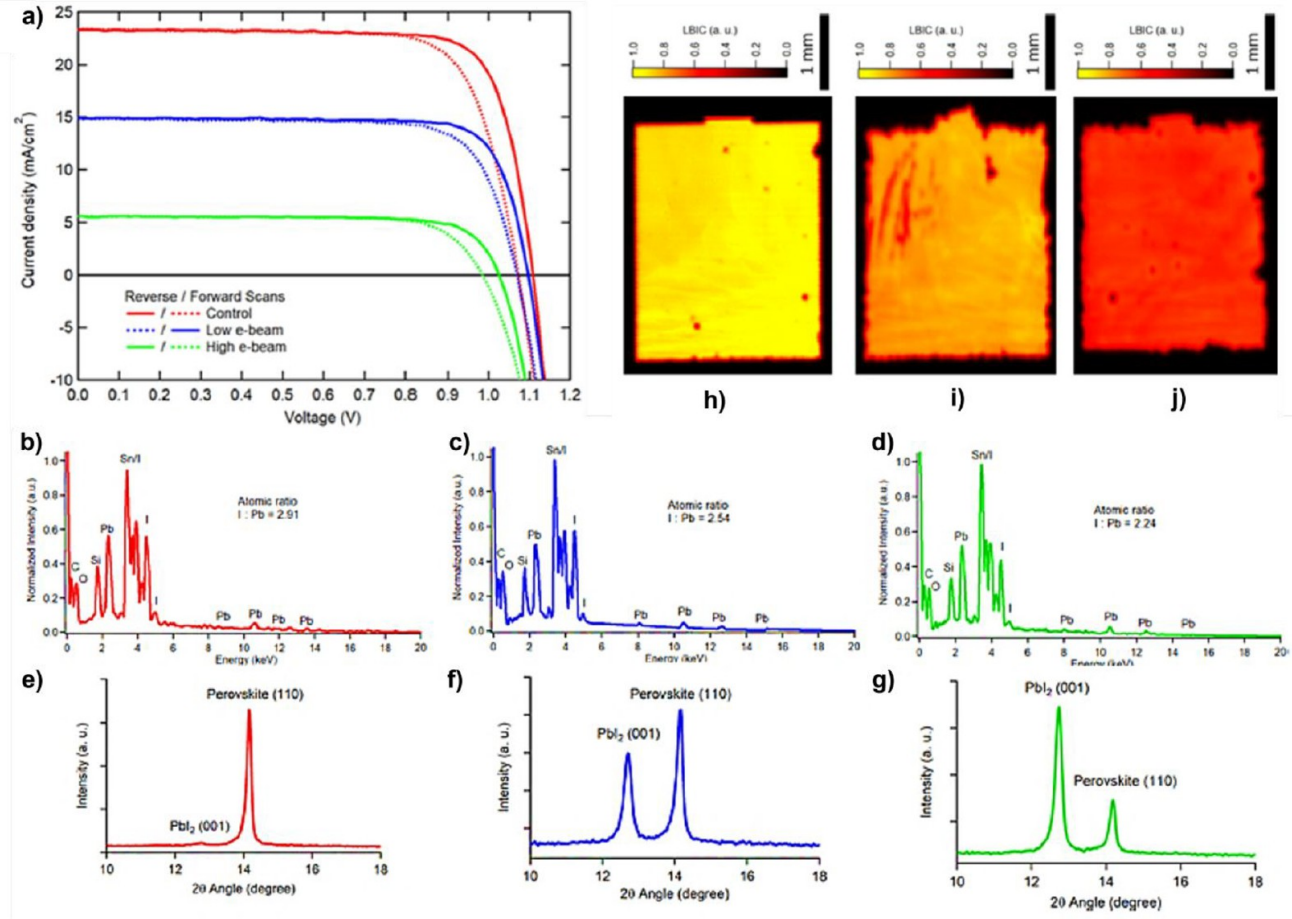
(a) Comparison of the *J*–*V* curves for reference (red lines) and bombarded with electrons having 1 MeV energy at a fluence of 1.3 × 10^13^ particles cm^–2^ (blue lines) and 1 × 10^15^ particles cm^–2^ (green lines). (b–d) Comparison of the EDX spectra measured for reference PSCs (red line) and PSCs bombarded with electrons having 1 MeV energy at a fluence of 1.3 × 10^13^ particles cm^–2^ (blue lines) and 1 × 10^15^ particles cm^–2^ (green lines). (h–j) Laser beam-induced current measurements for reference (h) and bombarded PSCs with electrons having 1 MeV energy at a fluence 1.3 × 10^13^ particles cm^–2^ (i) and 1 × 10^15^ particles cm^–2^ (j). Reprinted with permission from ref (84). Copyright 2019 American Chemical Society.

The effects of EB irradiation (with 1 MeV energy and accumulated doses of 1.3 × 10^13^ and 1 × 10^15^ particles cm^–2^) were also studied by Song *et al*.^84^ on n-i-p PSCs (with a MA_0.7_FA_0.3_PbI_3_ light harvester) with pristine PCE = 20.6%. Because of EB irradiation, such PCE decreased to 12.2% and 3.4% (for 1.3 × 10^13^ and 1 × 10^15^ particles cm^–2^, respectively), mainly because of a drastic drop of the *J*_SC_ while *V*_OC_ and FF remain almost unaffected (Figure 6a). Because EB irradiation causes the darkening of the glass substrate (as discussed previously for the case of PB irradiation), the authors considered the impact of this effect on the measured *J*_SC_ and PCE, revealing that the actual PCE retention of their devices is ∼81% and ∼56% (for 1.3 × 10^13^ and 1 × 10^15^ particles cm^–2^, respectively). Thus, there is still a performance loss associated with the perovskite layer. According to their energy-dispersive X-ray spectroscopy (EDX) and XRD results (Figure 6b–g), the irradiation process causes the partial decomposition of the perovskite into the photoinactive PbI_2_, as evidenced by the variation of the Pb:I ratio in EDX measurements and the appearance of the PbI_2_ contribution in the XRD spectra. Interestingly, in this work the authors report laser beam-induced current (LBIC) maps (Figure 6h–j) demonstrating that the drop in *J* photogeneration is homogeneously distributed throughout the whole device area, thus differing from water-induced degradation.^85^ These results seem very different from those by Miyazawa *et al*. (who reported a 90% retention of performance); however, it should be noted that the pristine performance of the devices built by Song *et al*. is much higher with respect to those by Miyazawa *et al*. (20% vs 5%). It is then clear that more research efforts are needed toward the assessments of the radiation stability of high-performance PSCs for two main reasons. First, it is difficult to address the degradation-induced losses of low-performance devices. Second, high-performance SCs are those required for practical use; thus, they are the most interesting for large-scale applications.

**Figure 7:**
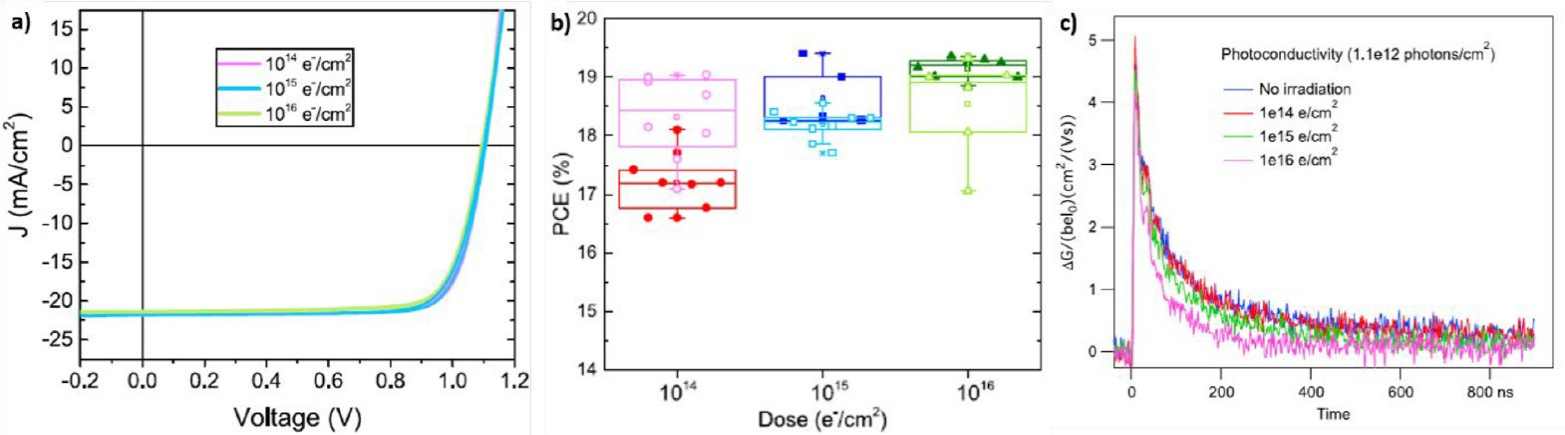
(a) Comparisons of the *J*–*V* curves for PSCs irradiated with electrons having 1 MeV energy at doses of 10^14^ particles cm^–2^ (pink line), 10^15^ particles cm^–2^ (cyan line), and 10^16^ particles cm^–2^ (green line). (b) Evolution of the PCE for PSCs before (empty symbols) and after (full symbols) irradiation at the aforementioned doses. Photoconductivity, as measured through time-resolved microwave conductivity experiments, of MAPbI_3_ thin films deposited on quartz. Reprinted with permission from ref (86). Copyright 2020 Wiley.

A similar study was presented by Pérez-del-Rey *et al*.,^86^ who investigated the radiation tolerance of MAPbI_3_-based PSCs (p-i-n architecture) to EB with 1 MeV energy at accumulated doses up to 10^16^ particles cm^–2^. The authors used both glass and quartz as substrate (because the former leads to performance losses due to radiation-induced darkening), and Figure 7a shows representative *J*–*V* curves obtained using the latter revealing a stable PCE ≈ 18.3%, up to 10^16^ particles cm^–2^. Quite interestingly, the authors highlight that basically all devices experience an increase of their PCE after irradiation at a dose of 10^14^ particles cm^–2^, while stable (or slightly lower) PCEs are observed for higher doses (Figure 7b). With the aim of understanding the effects of such radiation on the perovskite absorber, the authors conducted several characterizations of perovskite films (deposited on quartz), such as XRD, SEM, PL, and EQE, but no peculiar signs of degradation were observed. Finally, they performed time-resolved microwave conductivity (TRMC) analysis (Figure 7c). After photogeneration of electrons and holes, the conductivity decreases because of charge carrier recombination, mainly due to mono- and bimolecular processes. The decay of the photoinduced conductivity proceeds faster in the irradiated samples with respect to the pristine films (with a variation of the decay time from ∼40 to ∼55 ns, respectively), which indicates faster recombination dynamics due to the formation of trap states in the irradiated samples. From such kinds of measurements, the carrier diffusion length can be extracted, revealing quite interesting results: this quantity decreases from ∼0.8 μm in the nonirradiated sample to ∼0.65 μm in the sample irradiated with a dose of 10^16^ particle cm^–2^. Both values exceed the thickness of the MAPbI_3_ layer used in PSC fabrication, suggesting that charge carriers in irradiated MHPs still can reach the interfaces of the light harvester and thus be collected at the extraction layers.

**Figure 8:**
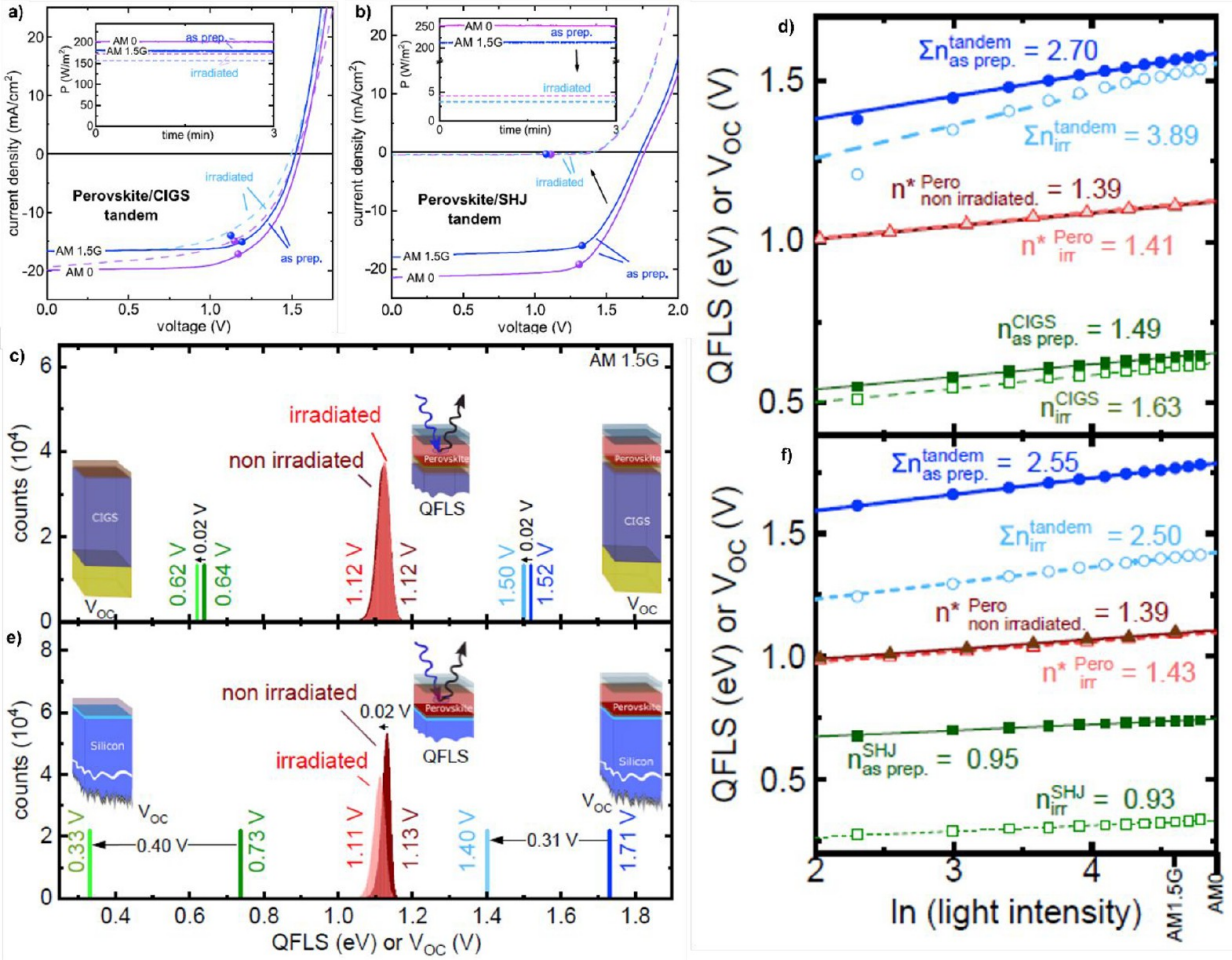
(a and b) Comparisons of the *J*–*V* curves for perovskite/CIGS (a) and perovskite/Si (b) tandem devices (solid lines for reference devices, dashed lines for irradiated solar cells). The maximum power point is indicated by the full circles, while the insets report the power output, at the maximum power point, as a function of time. (c and e) Comparisons of the quasi-Fermi level splitting and *V*_OC_ of perovskite/CIGS (c) and perovskite/Si (e) devices before and after the irradiation tests. (d and f) Quasi-Fermi level splitting as a function of the logarithm of the excitation intensity which allows the extrapolation of the ideality factors. Reprinted with permission from ref (41). Copyright 2020 Elsevier.

Another characteristic which makes MHPs intriguing materials for space PVs is the tunability of their band gap through engineering of their chemical composition,^30^ making them optimal candidates for the realization of tandem SCs. Two interesting candidates as bottom-cell light harvesters are Si and CIGS because perovskite/Si tandem devices are going to be commercially available for terrestrial applications,^87^ while perovskite/CIGS stacks can be realized in flexible configuration. Thus, a study by Lang *et al*.^41^ investigated the triple-cation MHPs (Cs_0.05_(MA_0.17_FA_0.83_)_0.95_Pb(I_0.83_Br_0.17_)_3_) as light harvester in both perovskite/Si and perovskite/CIGS devices (p-i-n configuration) and assessed their resistance to PB irradiation with 68 MeV energy at a dose of 10^12^ particles cm^–2^.^41^ In particular, the authors observed 85% and 1% of performance retention for perovskite/CIGS and perovskite/Si devices, respectively. The main degradation observed in the former architecture is a partial reduction of all the photovoltaic parameters which, however, remain high (as shown in Figure 8a).

Quite differently, in the case of perovskite/Si devices there is a drastic reduction of *J*_SC_ to ∼2% of its initial value (as reported in Figure 8b). With the aim of understanding the degradation mechanisms occurring in both architectures, the authors focused on *V*_OC_ losses and used PL measurements to extract the quasi-Fermi-level splitting (QFLS) of the whole stack (*i.e.*, perovskite/CIGS and perovskite/Si) and of the individual light harvesters (CIGS, Si, and perovskite). Figure 8c reports the values for the perovskite/CIGS architecture (blue lines), perovskite (red contribution), and CIGS (green lines) components. These data clearly show that the perovskite harvester does not exhibit any measurable variation of the QFLS due to PB irradiation, while the CIGS component shows a slight decrease of QFLS (0.02 V) which may be the responsible for the reduction of the QFLS observed in the complete perovskite/CIGS stack. Further information can be obtained by performing intensity-dependent *V*_OC_ measurements, which allow extracting the ideality factor (*n*) of the investigated materials. This quantity is directly related to the occurring charge carrier recombination mechanisms: *n* = 2 is associated with SRH recombination, *n* = 1 is ascribed to radiative electron–hole recombination, and *n* < 1 is due to Auger recombination.^88^ As shown in Figure 8d, the ideality factor of the perovskite absorber increases slightly (from 1.39 to 1.43) upon irradiation (still confirming the radiation resistance of MHPs) because of the formation of recombination centers. Similarly, the formation of recombination centers is responsible for the increase of *n* from 1.49 to 1.63 observed in the CIGS layer. The whole perovskite/CIGS stack shows an increase in *n* (which can be calculated as the sum of the ideality factors of the materials used in multijunction devices) from 2.70 to 3.89. Thus, the radiation damage experienced by the light harvesters does not explain the overall increase observed in *n* of the multijunction stack, suggesting that other processes contribute to the *V*_OC_ reduction in such an architecture. In particular, the authors suggest that interfacial recombination and radiation-damage induced to the other layers are the main factors. The same kind of analysis was performed also on the perovskite/Si device. Figure 8e reports the QFLS for this SC. Even in this case the perovskite QFLS remains almost unchanged while the Si absorber shows a high variation (0.40 V) which is in line with the losses shown by the perovskite/Si architecture. Interestingly, values of *n* (Figure 8f) change only slightly for both light harvesters (in particular, in Si *n* < 1 for both pristine and irradiated devices because of Auger recombination which is known to be the dominant recombination mechanism in Si-based SCs)^89^ and for the perovskite/Si stack (from 2.55 to 2.50), confirming once again the radiation tolerance of MHPs (and PSCs in general), as also demonstrated by other literature results.^90−93^

### Resistance to γ-rays and Neutrons

So far, the presented results concern the stability of MHPs with respect to bombardment by directly ionizing radiation. Space issues arise also because of indirectly ionizing radiation, such as γ-rays which have the highest penetration depth and cannot be stopped by shielding strategies. It was estimated that in 20 years of utilization, a space solar cell absorbs ∼10 000 Gy of γ radiation.^94^

**Figure 9:**
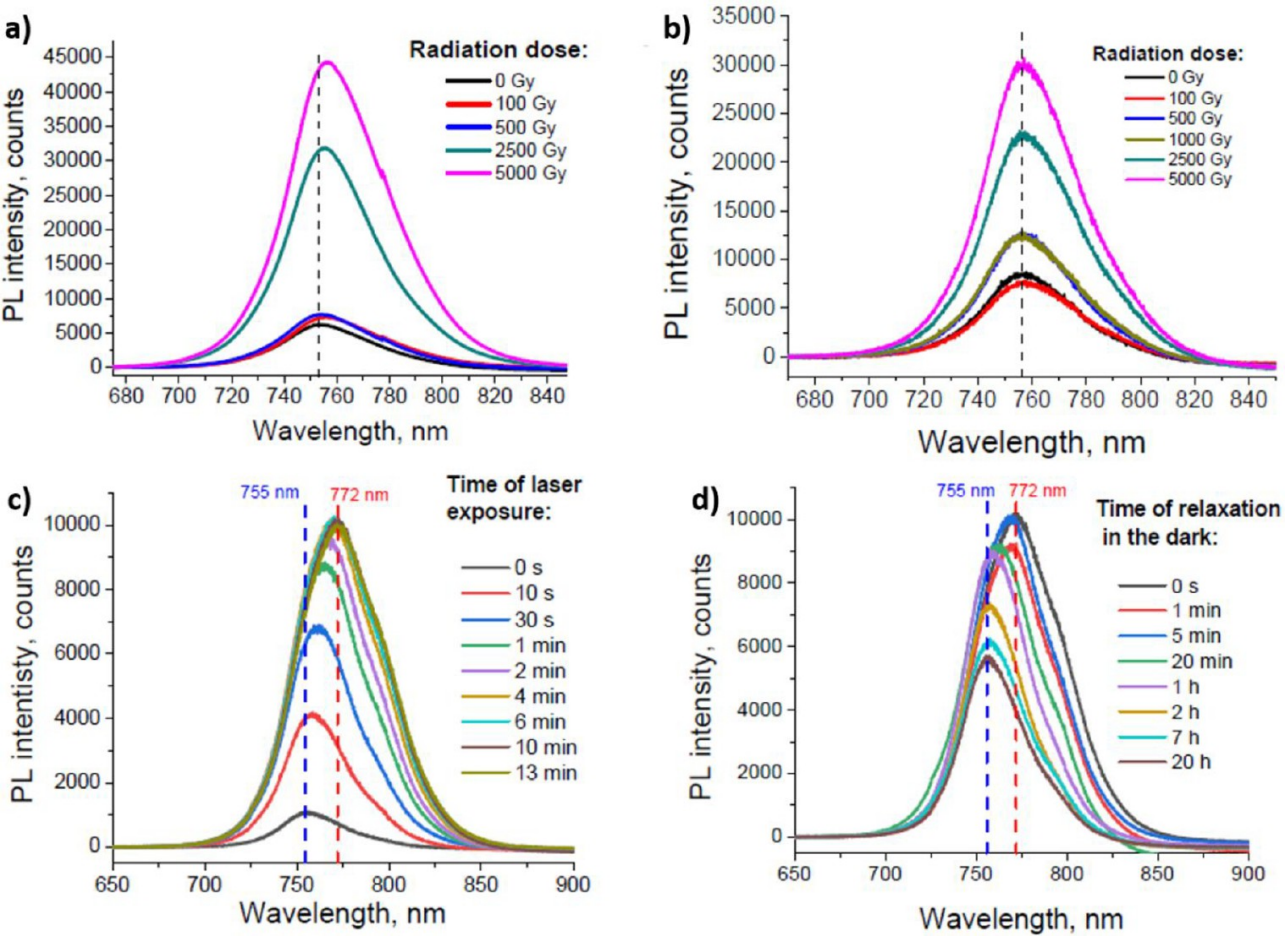
(a) Evolution of the PL emission of Cs_0.15_MA_0.10_FA_0.75_Pb(Br_0.17_I_0.83_)_3_ perovskite films at γ-ray doses up to 5000 Gy. (b) Comparison of PL spectra of Cs_0.15_MA_0.10_FA_0.75_Pb(Br_0.17_I_0.83_)_3_ perovskite films at γ-ray doses up to 5000 Gy, measured 2 weeks after the spectra reported in panel a. (c) PL spectra of Cs_0.15_MA_0.10_FA_0.75_Pb(Br_0.17_I_0.83_)_3_ perovskite films measured under green laser light (532 nm) illumination at different exposure time. (d) Time evolution of the PL emission of the samples, illuminated under the conditions reported in panel c and kept in the dark for times up to 20 h. Reprinted with permission from ref (95). Copyright 2019 American Chemical Society.

A very interesting work was reported by Boldyreva *et al*., who performed a study on the triple-cation MHP Cs_0.15_MA_0.10_FA_0.75_Pb(Br_0.17_I_0.83_)_3_ with the aim of addressing γ-ray-induced degradation (doses up to 5000 Gy).^95^ In particular, PL spectra (Figure 9) show an interesting evolution according to the radiation doses. The emission bands experience a red shift and the enhancement of their intensity, with respect to the nonirradiated sample, as the γ doses increase (Figure 9a). Quite interestingly, such features are reversible, *i.e.*, the same samples (analyzed after 2 weeks) show a reduced intensity with respect to the measurements reported in Figure 9a, while the red-shift completely disappears (Figure 9b). Such behavior cannot be attributed to the γ-radiation-induced formation of traps in the MHP crystal structure, as this scenario would imply the quenching of the PL signal. Quite differently, these results are consistent with those attributed to the so-called Hoke effect, which was observed on MHPs for the first time by Hoke *et al*.^96^ and reported frequently afterward, which is associated with halide-segregation with consequent formation of low band gap states. The Hoke effect is due to white-light illumination of MHPs and consists of a partially reversible sub-band gap emission feature in the PL spectra, which vanishes as samples are stored in the dark for several minutes.^97^ For this reason, Boldyreva *et al*. conducted a comparison between their results on γ-ray-irradiated samples and those obtained by illuminating triple-cation perovskite with a green laser (532 nm). As the time of laser exposure increases (up to 13 min), the PL signal intensity enhances and experiences a red-shift (Figure 9c).

Afterward, the samples were stored in the dark and measured again at time intervals ranging between 1 min and 20 h, leading to the temporal evolution of the PL spectra reported in Figure 9d (the authors illuminated the samples for short times <0.1 s during PL measurements). Notably, the red-shift of the PL contributions vanishes after 1 h of storing in dark condition, which explains the small red-shift observed in Figure 9a on samples irradiated with γ-rays. In fact, for those samples the authors had to wait ∼1 h before performing the PL characterizations because of safety issues related to the use of ionizing radiation. Finally, the authors characterized the performance of their devices, revealing PCE degradation from ∼10.2% (nonirradiated devices) to ∼7.0% (at 500 Gy doses) for the p-i-n architecture. However, the authors showed that these results are due also to the degradation of the electron-transporting layer (PC_61_BM), which poses another major challenge on MHP-based photovoltaics. Indeed, these devices comprise several constituents; thus, the radiation hardness of each component (charge carrier extraction layers, electrodes, *etc*.) must be properly assessed. Thus, Boldyreva *et al*. suggest that halide segregation represents a fundamental limiting factor concerning the use of mixed halide MHPs in solar cells for space use, hindering their use in such applications.

**Figure 10:**
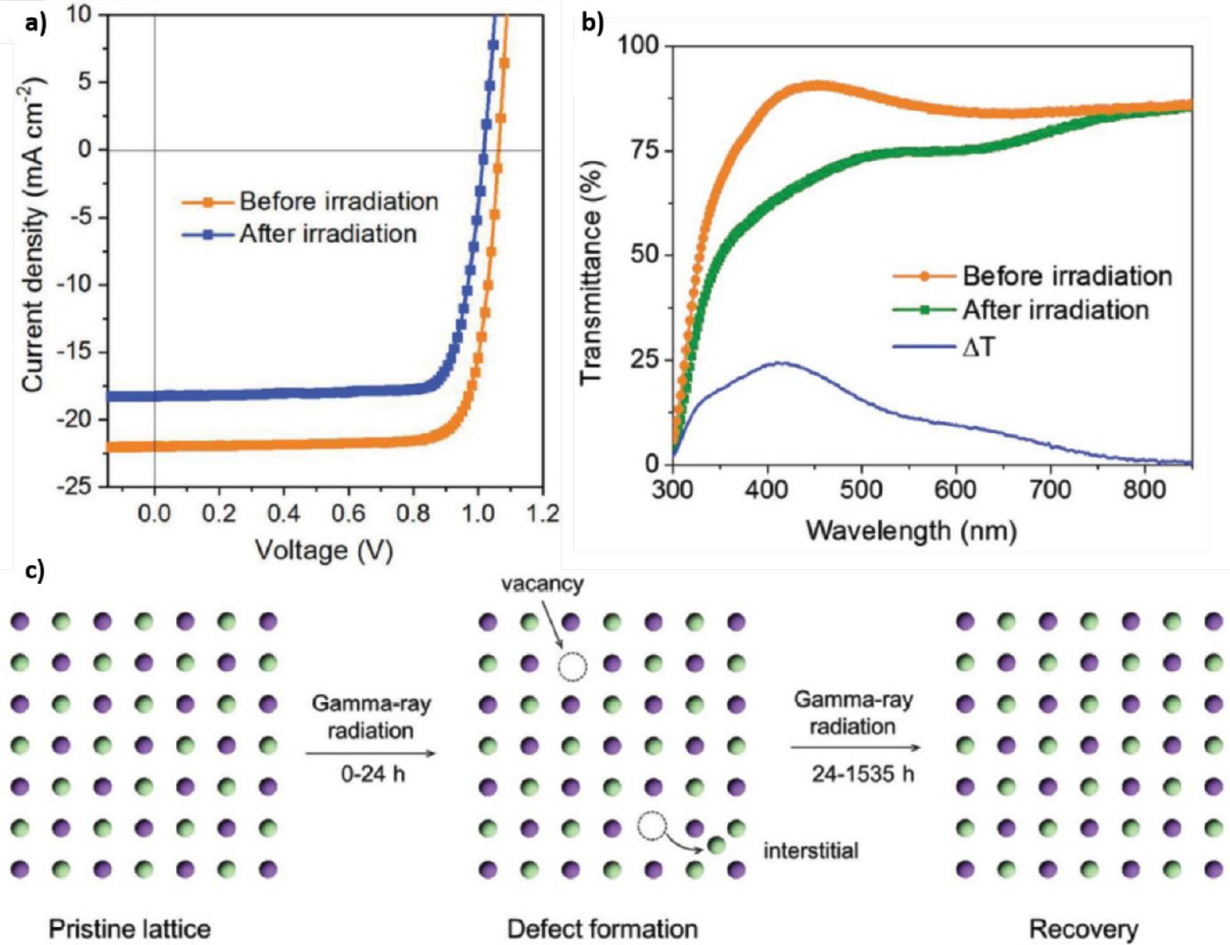
(a) Comparison of the *J*–*V* curves measured on Cs_0.05_MA_0.14_FA_0.81_PbBr_0.45_I_2.55_-based solar cells, before and after irradiation tests. (b) Variation of the transmittance spectrum of the glass/ITO substrate used for the fabrication of PSCs. The Δ*T* line indicates the transmittance loss associated with radiation-induced degradation. (c) Proposed self-healing mechanism of γ-ray-induced degradation in perovskites. Reprinted with permission from ref (98). Copyright 2018 Wiley.

Quite different results were obtained by Yang *et al*., who analyzed the photovoltaic performances of p-i-n solar cells using a triple-cation MHP as light harvester (Cs_0.05_MA_0.14_FA_0.81_PbBr_0.45_I_2.55_).^98^ In this study, the devices were illuminated by using both white light (at a 4.98 mW cm^–2^ intensity) and γ-rays for two reasons: simulating a more realistic space environment and studying the degradation effects due to the simultaneous presence of sunlight and high-energy radiation (which are known as main degradation sources of MHPs). Remarkably, in this work the authors performed a stability study for 1535 h of continuous light and γ-ray irradiation, reaching accumulated doses up to 23 000 Gy. Figure 10a reports the *J*–*V* curves measured before and after the stability test, revealing a reduction of the values of all the photovoltaic parameters, leading to an overall decrease of the PCE from the initial 18.80% to the final 14.95%. Interestingly, the *V*_OC_ exhibits a small variation (from 1.06 to 1.02 V) while *J*_SC_ shows a far higher reduction (from 21.98 to 18.26 mA cm^–2^). The authors attribute these effects to the darkening of the glass substrate because of the high accumulated γ-ray dose, as demonstrated also by several other works.^99−102^ In fact, radiation-induced damage causes a reduction of the transmittance of the glass from 90% to 50–75% in the visible range (as shown in Figure 10b). For this reason, the authors computed the photovoltaic parameters of their devices by considering the reduced transparency of the glass substrate: according to their results, the PCE of the p-i-n solar cell decreases from 18.80% to 18.20%. This is a quite surprising result, in contrast with that of Boldyreva *et al*., which underpins the high γ-ray radiation tolerance of MHPs (in this work, performance retention >96%) especially when compared with benchmark technologies such as Si-based photovoltaics, which retains <62% of the performances after accumulating doses ∼20 000 Gy.^103,104^ The authors attribute the radiation hardness of MHPs to self-healing of the perovskite crystal structure. In fact, γ-rays can displace atoms from their lattice sites, causing the formation of defects (such as vacancies, interstitials, *etc*.) during the first hours of irradiation. Afterward, because of the pronounced ion-migration behavior of MHPs, defects are recovered (Figure 10c).^105,106^

**Figure 11:**
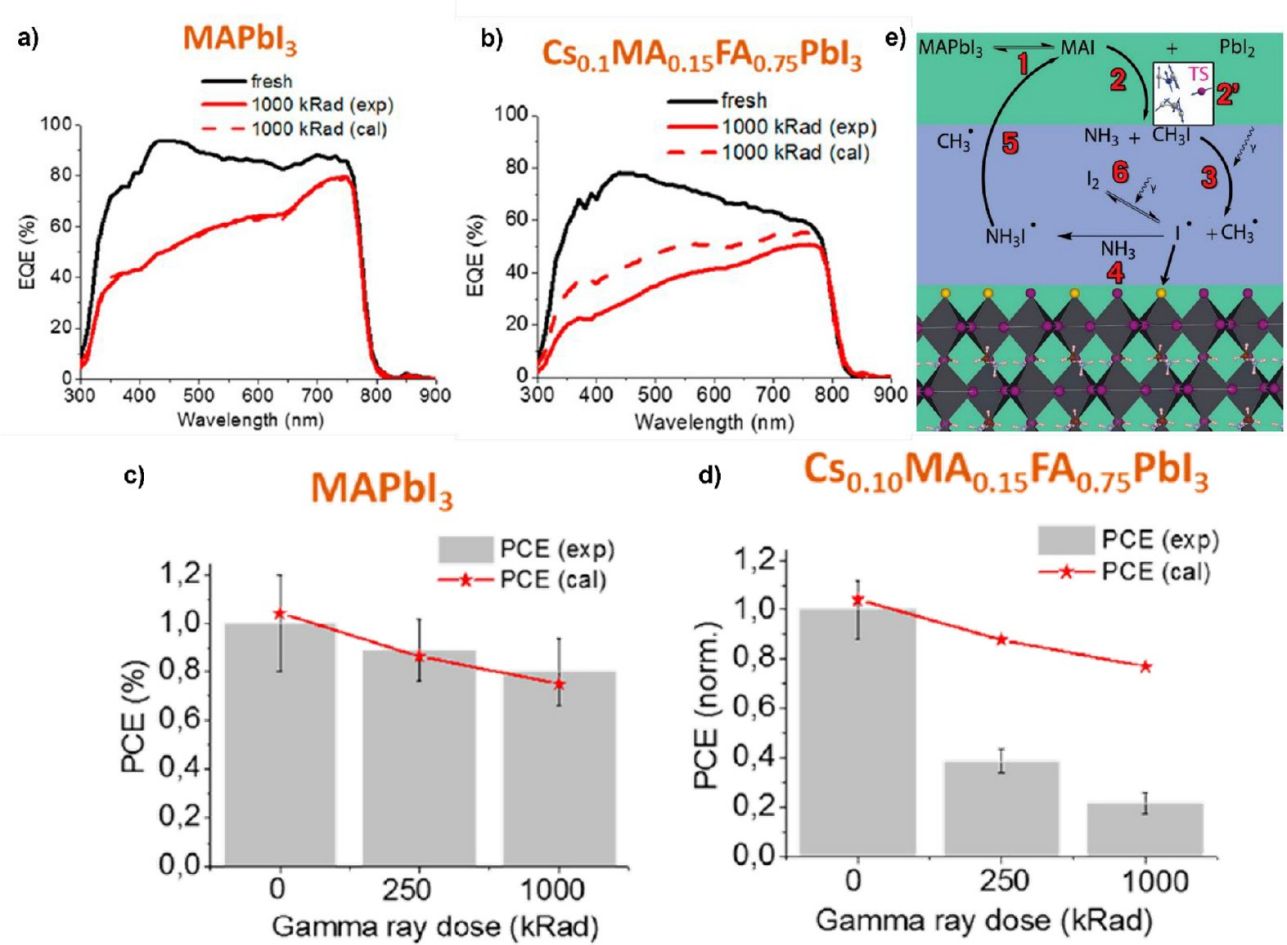
(a and b) Comparison of the EQE measurements on reference (black line) and irradiated (at 1000 kRad = 10000 Gy, red lines) MAPbI_3_ (a) and Cs_0.10_MA_0.15_FA_0.75_PbI_3_ (b). The solid red line refers to experimentally obtained data, while the dashed lines indicate simulated data, obtained by removing the effects due to γ-ray degradation induced on the glass/ITO substrate. (c and d) Evolution of the PCE of MAPbI_3_ (c) and Cs_0.10_MA_0.15_FA_0.75_PbI_3_ (b) devices as a function of the accumulated γ-ray dose. (e) Proposed mechanism for the self-healing of MAPbI_3_ perovskites. Details are reported in the text. Reprinted with permission from ref (107). Copyright 2020 American Chemical Society.

In a later study, Boldyreva *et al*.^107^ investigated the γ-ray resistance (with doses up to 5000 Gy) of several MHPs that contain only one halide component: MAPbI_3_, MAPbBr_3_, Cs_0.15_FA_0.85_PbI_3_, Cs_0.10_MA_0.15_FA_0.75_PbI_3_, CsPbI_3_, and CsPbBr_3_. Both XRD and XPS characterizations show that MAPbI_3_ suffers from a partial decomposition due to the loss of MAI and consequent formation of PbI_2_ regions (for doses >1000 Gy) only at the surface of the MHP. A similar behavior was observed in CsPbI_3_, but for this case, metallic lead (Pb^0^) was detected rather than PbI_2_ (this is due to the absence of the volatile MAI component). The remaining investigated compositions revealed no sign of γ-ray-induced degradation. Finally, the PL spectra of such samples reveal no appreciable differences in CsPbBr_3_ and CsPbI_3_; quenching effects in MAPbI_3_ (because of the decomposition of the crystal structure that leads to the formation of shallow defects); decrease of the emission intensity for doses up to 500 Gy (and stable for higher values) in MAPbBr_3_; increase of the PL intensity in Cs_0.15_FA_0.85_PbI_3_, Cs_0.10_MA_0.15_FA_0.75_PbI_3_. For the latter cases, the authors suggest a similar process as the one discussed for the mixed-halide triple-cation perovskite: phase segregation causes the formation of crystalline domains with increased structural order that enhances the PL quantum yields. Furthermore, the authors performed UV–vis characterization of their MHPs and attributed the slight observed differences to darkening of the glass substrate due to the formation of color centers which vary the transmittance of the glass. For this reason, the authors measured the external quantum efficiency (EQE) of their fresh nonirradiated solar cells and at 10 000 Gy; then they calculated the EQE by considering the glass darkening as the only effect induced by γ-ray degradation. It is worth noting that the MAPbI_3_ sample (Figure 11a) shows no difference between the experimental and calculated EQE at 10 000 Gy, meaning that the only degradation mechanism occurring concerns only the glass substrate. Quite differently, the other MHPs show deviations from this ideal situation, in particular for the case of Cs_0.10_MA_0.15_FA_0.75_PbI_3_ (Figure 11b); thus, other degradation pathways are activated for this type of light harvester. Such results are further confirmed by the PCE characterizations showing good agreement between the experimental and calculated values for MAPbI_3_ (Figure 11c), whereas huge differences are observed for the case of Cs_0.10_MA_0.15_FA_0.75_PbI_3_ (Figure 11d). The authors attribute the γ-ray resistance of MAPbI_3_ to a rapid self-healing process (Figure 11e), based on and supported by other literature results. The degradation of MAPbI_3_ leads to the formation of MAI and PbI_2_ (step 1, Figure 11e), and the former then converts mainly into NH_3_ and CH_3_I (step 2, Figure 11e). Because the C–I bond is weak, γ-rays can break it, leading to the formation of CH_3_^+^ and I^–^ (step 3, Figure 11e). The I^–^ anion can then follow three different pathways: passivating an iodine vacancy (yellow sphere, step 4, Figure 11e); forming a I_2_ molecule which can be broken by γ-rays (step 6, Figure 11e) or react with NH_3_, forming NH_3_I^–^. In this latter case, the NH_3_I^–^ can react with CH_3_^+^ cation, thus forming again MAI which can eventually react with PbI_2_ leading again to the MAPbI_3_ perovskite. Thus, for the authors, the γ-ray resistance of MAPbI_3_ arises because of the reversible formation of MAI, the chemical reactions favored by γ-rays, and the passivating effects of the iodine vacancies which can occur in MHPs characterized by such chemical composition. Although this model successfully explains and links many aspects related to γ-ray-induced effects, we want to highlight that such conclusions are discussed by considering the temperature as 298 K, *i.e.*, room temperature. Thus, it is possible that some reaction pathways are hindered (or facilitated) when real space environments are considered because temperatures can reach great extremes (−150 to +180 °C).

**Figure 12:**
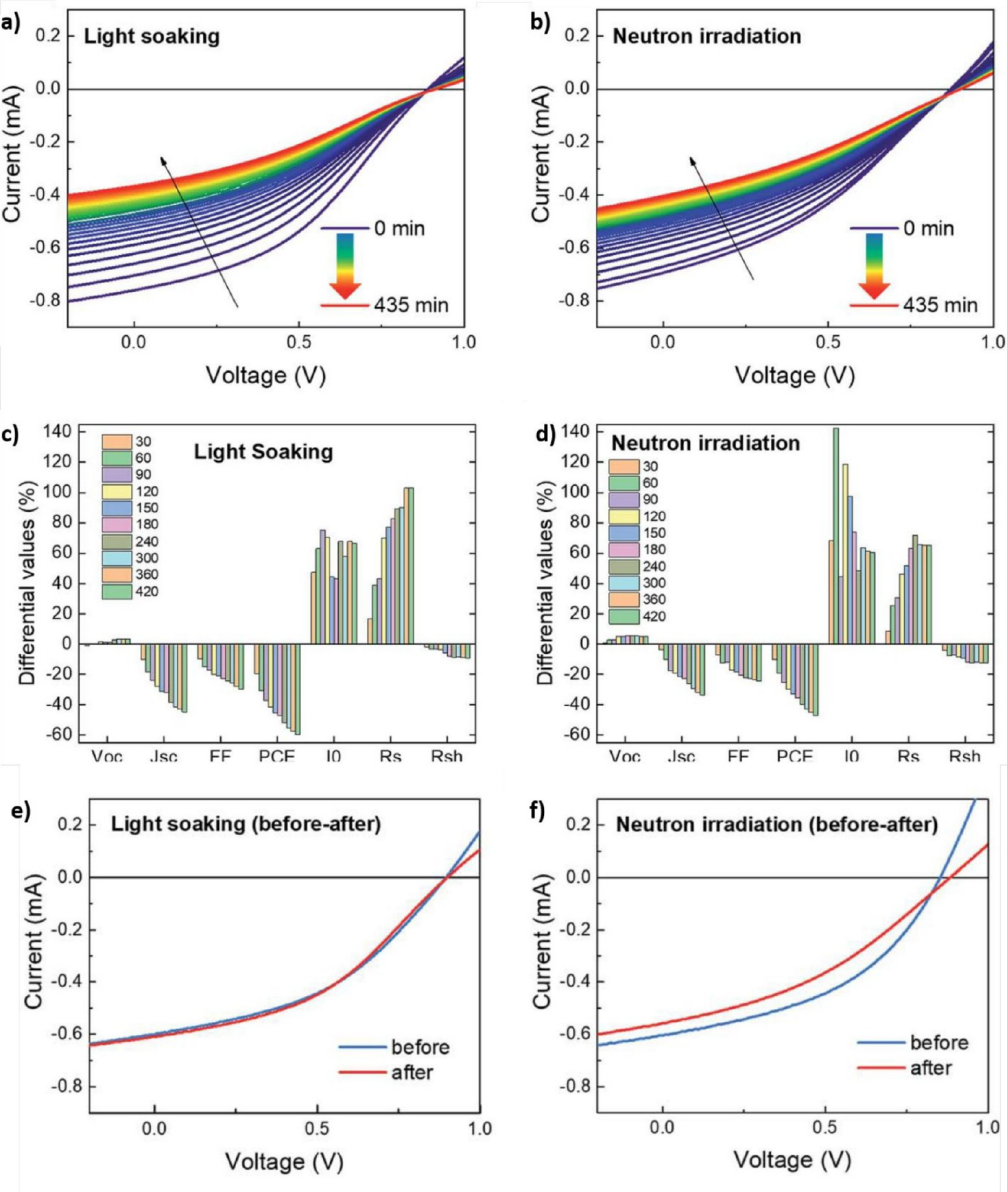
(a) Time evolution of the *I*–*V* curves for illuminated nonirradiated and (b) for neutron-irradiated illuminated MAPbI_3–*x*_Cl_*x*_-based p-i-n PSCs (measurements taken every 15 min). (c) Evolution of the PV parameters, *I*_0_, *R*_S_, and *R*_SH_ for illuminated nonirradiated and (d) for neutron-irradiated illuminated devices. (e) *I*–*V* characteristics taken before (blue line) and after (red line) the set of measurements reported in panel a for illuminated nonirradiated devices. (f) *I*–*V* characteristics taken before (blue line) and after (red line) the set of measurements reported in panel b for illuminated neutron-irradiated devices. Reprinted with permission from ref (110). Copyright 2019 Royal Society of Chemistry.

Lastly, it is worth discussing the resilience of MHPs to neutron bombardment. Fast neutrons (*i.e.*, with energy >10 MeV) are generated through collision between the incoming plasma (or cosmic rays) with the atmosphere’s constituents or with materials making up spacecraft.^108,109^ Assessing the resistance of materials to neutrons is fundamental as the accumulated dose of such particles, in one year, can be as high as 2.8 × 10^11^ particles cm^–2^ at the International Space Station orbit (with an energy spectrum varying between 10^–1^ to 10^11^ eV).^109^ To the best of our knowledge, currently there are only two studies in the literature that investigated the consequences of fast neutron bombardment on MHPs. The first work, by Paternò *et al*., focused on p-i-n PSCs (with a MAPbI_3–*x*_Cl_*x*_ absorber), using the spallation source available at the ISIS facility.^110^ Notably, spallation sources use neutrons produced through the bombardment of targets with accelerated protons, so the experiment fairly reproduces the space environment (in this study, a fluence of 1.5 × 10^9^ particles cm^–2^ s^–1^ was used, corresponding to ∼80 years of exposure to fast neutrons at the International Space Station are replicated). The authors report *in operando* measurements in a time range between 0 and 435 min, comparing the results obtained for illuminated (nonirradiated) and illuminated (irradiated) samples (panels a and b of Figure 12, respectively) to discern light-induced effects from those due to neutron bombardment.

Interestingly, more pronounced PCE losses are observed for the nonirradiated devices than the neutron irradiated PSCs (60% and 45% respectively, Figure 12c,d). To obtain further insights in their results, the authors focused on the analysis of the diode equation, typically used for the description of SCs:^111,112^
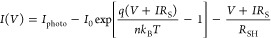
where *I* and *V* are the current and the voltage of the cell, respectively; *I*_photo_ is the photogenerated current; *I*_0_ is the leakage current; *q* is the elementary charge; *R*_S_ and *R*_SH_ are the series and shunt resistance, respectively; *k*_B_ is the Boltzmann constant; *T* is the temperature (in absolute units); and *n* is the ideality factor. In particular, the authors characterized the evolution of the photovoltaic parameters and of *I*_0_, *R*_S_, and *R*_SH_ (Figure 12c,d). In fact, the latter quantities bear several pieces of information about charge-carrier dynamics within light-harvester materials: *I*_0_ represents the thermal equilibrium recombination current, *R*_S_ corresponds to the resistance experienced by charge carriers during their motion within the light-harvester and at the interfaces with the charge extraction layers, while *R*_SH_ relates to the presence of short-circuit paths in the device.^112,113^ The results show an increase of *I*_0_ (associated with a more pronounced recombination dynamics) and an increase of *R*_S_ accompanying a decrease in *R*_SH_ for both irradiated and nonirradiated cases. According to the authors, these observations point toward the formation of trap states in the light-harvester and the electrodes. With the aim of discerning between light- and neutron-induced degradation, the authors compared the *J*–*V* curves of their devices before and after the light and neutron exposure (Figure 12e,f). For the case of nonirradiated samples, the *J*–*V* curves remain essentially the same (Figure 12e), confirming the practical reversibility of light-induced degradation in PSCs.^114^ Quite differently, neutron-bombarded devices experience variations in their *J*–*V* curves (Figure 12f), suggesting that neutron-induced losses are not reversible. As such, the authors suggest that neutrons cause the irreversible displacements of atoms from their crystalline sites, leading mainly to the formation of Frenkel defects (*i.e.*, pairs of vacancy and interstitial defects).^115^ Although it is not possible to discern which layer suffers from the highest losses, this work shows that PSCs can withstand neutron bombardment. We point out that the devices presented in this work have a starting PCE of ∼6%; thus, further investigations are needed to thoroughly understand neutron-induced losses in PSCs (especially in the MHP layer) and to conduct such tests in high-PCE devices.

A step toward this direction was recently taken by De Rossi *et al*., who performed an investigation on neutron-bombarded flexible PSCs (where a Cs_0.06_FA_0.78_MA_0.16_Pb(I_0.84_Br_0.16_)_3_ light harvester was used) with the aim to compare the stability of two different hole-transporting materials: 2,2(7,7)-tetrakis(*N*,*N*-dipmethoxyphenylamine)9,9(-spirobifluorene) (spiro-OMeTAD) and benzothiadiazole-modified poly(3-hexylthiophene-2,5-diyl) (BTD-P3HT).^116^ According to their results, although BTD-P3HT-based devices suffered from a higher PCE loss (from ∼9.1% to ∼6.37%) under a fluence of 10^9^ neutrons cm^–2^, the overall performance loss (*i.e.*, by considering also *J*_SC_ and *V*_OC_ values) was lower with respect to the solar cells where spiro-OMeTAD was used (which showed a PCE drop from ∼9.2% to ∼8.74%). The authors infer that BTD-P3HT can sustain higher neutron doses with respect to the spiro-OMeTAD, but the presence of a nonoptimal interface between the perovskite absorber and the hole-transporting materials leads to increased performance losses. Thus, with further research and an optimized modification of the P3HT polymer, the authors suggest that it can be possible to find a suitable hole-transporting material for the realization of PSCs for space applications.

## Space Environment Impact on the Design of Perovskite Solar Cells

Because radiation is not the only source of performance losses in PV devices for space applications, herein we present a perspective about the possible design and implementations for PSCs that should be able to fulfill the requirements in terms of device stability and reliability (considering the real space environment) together with the main constraints in terms of low weight and technology scalability.

As a first consideration, because space transportation is very expensive (today the cost to launch a satellite into orbit is of $20 000 per kg),^117^ one of the primary goals of space agencies is to realize light and consequently cheaper satellites. If, on one hand, the specific power of PSCs was estimated as one of the highest of all PV technologies (23 W g^–1^), the device substrate plays a crucial role in maintaining the overall device weight as low as possible. In this regards, flexible substrates (such as PET-ITO) or rigid glass-ITO with reduced thickness, are the preferential starting point for device fabrication. However, as previously mentioned, glass usually suffers from radiation-induced darkening that could affect the PSCs’ performance duration. Therefore, quartz substrates are preferred to be employed because the specific weight of quartz is usually lower than that of common glass. Moreover, quartz could be successfully made conductive by ITO sputtering deposition or by depositing single-layer graphene by a chemical vapor deposition (CVD) technique.^118,119^ Indeed, more recently, bidimensional (2D) materials such as graphene and related 2D materials were extensively employed in PV technologies.^120^ In particular, the replacement of transparent conductive oxide (TCO) by cheaper materials is currently a hot topic in the scientific community’s efforts devoted to developing reproducible and scalable graphene deposition processes. Regarding the use of flexible PET substrates, if on one hand this allows the possibility to conceive roll-up solar panels reducing the bulk and weight of a satellite’s power system, on the other hand it does not allow the use of temperatures above 250 °C during the device realization, limiting the choice of the perovskite absorber, charge-transporting layers (CTLs), and panel lamination process.

Once the substrate is chosen, the device architectures that can be implemented are n-i-p or p-i-n based on planar or mesoscopic configuration. Recently, the highest power conversion efficiency reached for PSCs employed a mesoscopic structure based on a TiO_2_ scaffold.^121^ However, temperatures above 460 °C are required for depositing compact TiO_2_ by spray pyrolysis deposition and to accomplish the mesoporous TiO_2_ sintering process.

Moreover, the decreased density of the atmosphere (or its absence, depending on the altitude) introduces another factor that can affect the stability of space PV technologies, *i.e.*, the presence of UV radiation. This is particularly important in PSCs because UV instability of these devices represents one of the main factors hindering their commercial spread. In fact, UV radiation can damage PSCs containing TiO_2_ or organic transporting layers.^5^ The former affects the performance of PSCs because of its photocatalytic properties which, activated by UV radiation, cause the formation of halide species (I_2_, Cl_2_, and Br_2_) and consequently the irreversible degradation of the MHP structure.^122−124^ For these reasons, several strategies have been proposed and are still the subject of intense research to overcome this issue, such as (i) the use of down-converting materials (to convert UV photons into visible photons);^125,126^ (ii) the addition of an interlayer between TiO_2_ and MHPs;^127,128^ (iii) the replacement of TiO_2_ with other materials;^129,130^ and (iv) the reduction of the photocatalytic activity of TiO_2_ by doping or by using UV filters.^130,131^

Thus, in order to make the PSC fabrication process as easy and cheap as possible, planar structure should be considered as the first choice. In particular, both p-i-n and n-i-p structures have been already tested under high-energy cosmic radiation, including protons, electrons, and γ-rays as discussed herein and summarized in Table 1.

**Table 1:** Values of PCE for PSCs Tested under High-Energy Charged Particles and AM 1.5G (100 mW cm^–2^) Conditions^a^

solar cell architecture	energy	maximum dose	initial PCE	final PCE	ref
Electron Radiation					
ITO/TiO_2_/**FAPbI**_**3**_/Spiro-OMeTAD/Ag	1 MeV	10^16^ particles cm^–2^	12.2%	10.98%	(132)
glass/FTO/SnO_2_/**C**_**60**_**-SAM/MA**_**0.7**_**FA**_**0.3**_**PbI**_**3**_/Spiro-OMeTAD/Ag	1 MeV	10^15^ particles cm^–2^	19.2%	3.4%	(84)
glass/FTO/TiO_2_/**MAPbI**_**3–*x***_**Cl**_***x***_/P3HT/Au	1 MeV	10^16^ particles cm^–2^	4.8%	∼4.5%	(70)
glass/FTO/TiO_2_/**Cs**_***x***_**FA**_**0.85**_**MA**_**0.15**_**Pb(I**_**0.85**_**Br0.15)**_**3**_/P3HT/Au	1 MeV	10^16^ particles cm^–2^	4.4%	∼4.3%	(70)
Proton Radiation					
ITO/NiO/**MAPbI**_**3**_/PCBM/Ag	50 keV	10^12^ particles cm^–2^	12.3%	5.16%	(132)
glass/ITO/PEDOT:PSS/**CH**_**3**_**NH**_**3**_**PbI**_**3**_/PCBM/BCP/Ag	68 MeV	10^13^ particles cm^–2^	12.1%	4.84%	(71)
quartz/AZO/SnO_2_/**Cs**_**0.05**_**(MA**_**0.17**_**FA**_**0.83**_**)**_**0.95**_**Pb(I**_**0.83**_**Br**_**0.17**_**)**_**3**_/ Spiro-OMeTAD/Au	150 keV	10^15^ particles cm^–2^	15%	3%	(117)
ITO/PEDOT:PSS/**MAPbI**_**3**_/PC_61_BM/BCP/Ag	68 MeV	10^13^ particles cm^–2^	4.7%	5.7%	(72)
glass/FTO/TiO_2_/**MAPbI**_**3–*x***_**Cl**_***x***_/P3HT/Au	50 keV	10^15^ particles cm^–2^	4.8%	∼5.3%	(70)
glass/FTO/TiO_2_/**Cs**_***x***_**FA**_**0.85**_**MA**_**0.15**_**Pb(I**_**0.85**_**Br**_**0.15**_**)**_**3**_/P3HT/Au	50 keV	10^15^ particles cm^–2^	4.4%	3.5%	(70)
quartz/ITO/PTAA/**Cs**_**0.05**_**MA**_**0.17**_**FA**_**0.83**_**Pb(I**_**0.83**_**Br**_**0.17**_**)**_**3**_/C_60_/ BCP/Cu	68 MeV	10^12^ particles cm^–2^	18.8%	17.86%	(7)
γ-Rays Radiation					
glass/ITO/SnO_2_/**FA**_**0.945**_**MA**_**0.025**_**Cs**_**0.03**_**Pb(I**_**0.975**_**Br**_**0.025**_**)**_**3**_/spiro-OMeTAD/Ag	1.25 MeV	500 krad (Si)	19.03%	∼14%	(99)
ITO/PTTA/**Cs**_**0.05**_**FA**_**0.81**_**MA**_**0.14**_**PbI**_**2.55**_**Br**_**0.45**_/C_60_/BCP/Cu		2.3 Mrad	18.8%	14.95%	(98)
PEN/ITO/SnO_2_/**FA**_**0.945**_**MA**_**0.025**_**Cs**_**0.03**_**Pb(I**_**0.975**_**Br**_**0.025**_**)**_**3**_/spiro-OMeTAD/Ag	1.25 MeV	500 krad (Si)	16.08%	13.63%	(133)
glass/ITO/PEDOT:PSS/**Cs**_**0.15**_**MA**_**0.10**_**FA**_**0.75**_**Pb(Br**_**0.17**_**I**_**0.83**_**)**_**3**_/PC_61_BM		500 Gy	>10%	∼6%	(95)
ITO/SnO_2_/PCBA/**Cs**_**0.1**_**MA**_**0.15**_**FA**_**0.75**_**PbI**_**3**_/PTA/MoO_3_/Al		1000 krad	∼13%	∼3%	(107)
ITO/SnO_2_/PCBA/**Cs**_**0.15**_**FA**_**0.85**_**PbI**_**3**_/PTA/MoO_3_/Al		1000 krad	∼11%	∼7%	(107)
ITO/SnO_2_/PCBA/**MAPbBr**_**3**_/PTA/MoO_3_/Al		1000 krad	∼5%	∼2%	(107)
ITO/SnO_2_/PCBA/**CsPbI**_**3**_/PTA/MoO_3_/Al		1000 krad	∼6.5%	∼3%	(107)
ITO/SnO_2_/PCBA/**CsPbBr**_**3**_/PTA/MoO_3_/Al		1000 krad	∼3%	∼2%	(107)
ITO/SnO_2_/PCBA/**MAPbI**_**3**_/PTA/MoO_3_/Al		1000 krad	∼10%	∼9%	(107)
Neutron Radiation					
ITO/PEDOT:PSS/**MAPbI**_**3**_**(Cl)**/PCBM/Al	10 MeV	1.5 × 10^9^ particles cm^–2^	6	5.16	(110)
PET/ITO/SnO_2_/**Cs**_**0.06**_**FA**_**0.78**_**MA**_**0.16**_**Pb(I**_**0.84**_**Br**_**0.16**_**)**_**3**_/spiro-OMeTAD/Au		10^9^ particles cm^–2^	∼9.2%	∼6.37%	(116)
PET/ITO/SnO_2_/**Cs**_**0.06**_**FA**_**0.78**_**MA**_**0.16**_**Pb(I**_**0.84**_**Br**_**0.16**_**)**_**3**_/P3HT/Au		10^9^ particles cm^–2^	∼9.1%	∼8.74%	(116)

a Some of the PCE values are calculated by using the remaining factors and the initial value of PCEs or estimated based on the evolution curve of the PCEs as function of the particle dose; thus, these PCE values may be under- or overestimated.

When choosing among p-i-n and n-i-p architectures, three main factors should be taken into account: (i) the stability of the employed CTLs/electrodes in the space environment; (ii) the temperature constraints for the perovskite layer processing imposed by the used CTLs; (iii) the constraints in terms of applied temperature and pressure dictated by the encapsulation and lamination procedures once the full cell is realized.

**Figure 13:**
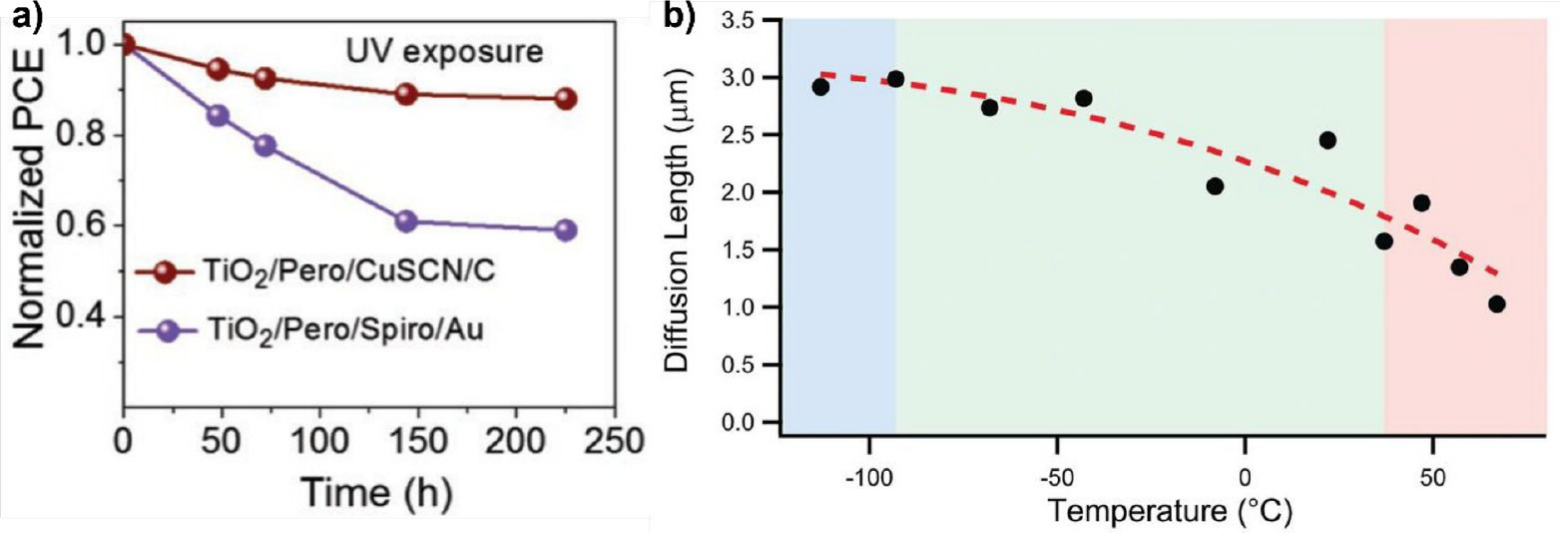
(a) Comparison of the PCE retention under UV illumination for PSCs using spiro/Au or CuSCN/C ad hole-transporting materials and electrodes. Reprinted with permission from ref (134). Copyright 2019 Wiley. (b) Temperature evolution of charge carrier diffusion length of MAPbI_3_ thin films, as obtained by using data from time-resolved PL and optical-pump THz-probe experiments. Reprinted with permission from ref (142). Copyright 2015 Wiley.

Indeed, it was shown that the organic transporting layers can also contribute to the UV instability of PSCs. In particular, a study by Arora *et al*. showed that the substitution of the standard 2,2(7,7)-tetrakis(*N*,*N*-dipmethoxyphenylamine)9,9(-spirobifluorene) (Spiro-OMeTAD) organic hole-transporting material with a CuSCN selective layer results in an increase of the PCE retention (during UV stress tests) from ∼60% to >80%, respectively (Figure 13a).^134^ Thus, structures based on an inorganic CTL have more chances to survive within a real space environment while allowing for higher temperature to be applied during cell/module encapsulation and panel lamination. Moreover, the use of an inorganic CTL, able to sustain temperatures higher than 300 °C during the realization of the perovskite absorber on top of it, could allow the deposition of an inorganic perovskite layer that could be a good choice when considering the high vacuum conditions of the space environment. As a matter of fact, high vacuum conditions can cause outgassing of volatile materials that can redeposit because of condensation on colder surfaces. For this reason, outgassing tests (such as the ASTM Standard E1559)^135^ are usually performed to assess the amount of volatile mass in a device and to evaluate outgassing contamination that can affect other constituents of the spacecraft.^136^ Several research contributions showed that MHPs can release compounds already at 10^–4^ Pa, such as CH_3_NH_2_, HI, CH_3_I, CHNH_2_, HI, *etc*.^137,138^ These effects are mitigated in complete devices, as MHPs are sandwiched between CTLs.^137^ Moreover, it was shown that vacuum-induced degradation depends on both MHP composition and encapsulation strategies;^70,139−141^ thus, several aspects must be taken into account for the efficient suppression of vacuum degradation pathways.

Furthermore, thermal cycling stress tests are fundamental to assess the stability of materials with respect to such factors. One of the standard tests that must be passed by materials used for PV applications is the AIAA S-111A-2014 (from the American Institute of Aeronautics and Astronautics), which uses temperatures between −185 °C and +150 °C.^143^ For the case of PSCs, thermal cycling can affect the performance in three main ways. First, because PSCs are heterojunction devices, thermal expansion/contraction of the different layers can cause the delamination of the SC because of differences in the expansion coefficients. Second, temperature variations can induce phase transitions between various polymorphs of the perovskite crystal structure.^144^ For example, MAPI shows a cubic structure above ∼ +57 °C, a tetragonal phase between ∼ +57 °C and ∼ −113 °C, and finally an orthorhombic phase below ∼ −113 °C.^28,145^ Thus, it is fundamental to determine if such transitions can affect the PV performance of PSCs. Temperature-dependent measurements can provide a useful tool to this aim, as shown by Milot *et al*., who investigated the charge-carrier dynamics in MAPI thin films.^142^ Their results show that in the temperature range between −93 °C and +67 °C the charge-carrier diffusion length is always higher (>1 μm, Figure 13b) with respect to typical thickness of the perovskite layer (∼500 nm), thus allowing the efficient extraction of electrons and holes. However, it is well-known that MAPI shows signs of decomposition already at +85 °C, because of the release of organic components (CH_3_NH_2_); thus, it may not be a suitable candidate for those applications where temperatures higher than this threshold are reached.^144,146^ Fortunately, chemical engineering of the MHP structure can potentially solve this problem by using all-inorganic Cs-based MHPs^147−150^ and low-dimensional (2D or quasi-2D) MHPs.^24,151^ Concerning the low-temperature regime, MA- and FA-based MHPs exhibit phase transitions^137,152−154^ while mixed-cation MHPs are more stable.^155^ Moreover, Yang *et al*. demonstrated that all-inorganic MHPs do not experience any phase transition down to 4 K.^156^

**Figure 14:**
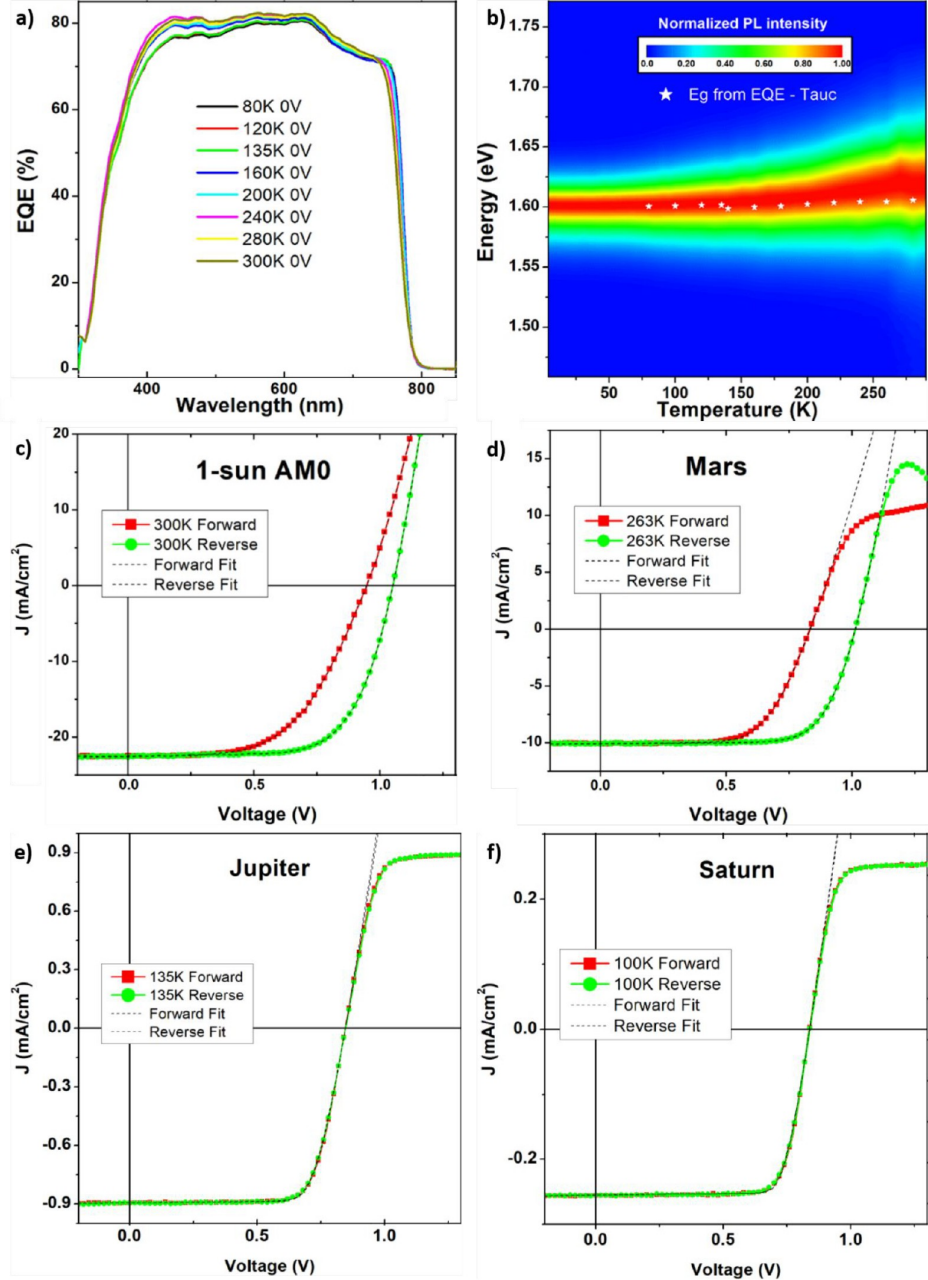
(a and b) Temperature evolution of the EQE (a) and PL (b) of (FA_0.79_MA_0.16_Cs_0.05_)_0.97_Pb(I_0.84_Br_0.16_)_2.97_-based solar cells. (c–f) *J*–*V* curves measured at temperature and intensity conditions typical of low-Earth orbit (c), Mars (d), Jupiter (e), and Saturn (f). Reprinted with permission from ref (157). Copyright2018 American Chemical Society.

Regarding PSC behavior at low-temperature conditions, it is worth mentioning a study by Brown *et al*., who investigated the potential use of n-i-p PSCs (based on a (FA_0.79_MA_0.16_Cs_0.05_)_0.97_Pb(I_0.84_Br_0.16_)_2.97_ active layer) in low-intensity–low-temperature (LILT) conditions.^157^ At these conditions, temperatures as low as 4 K can be reached; thus, the authors measured both EQE and PL of the PSCs (Figure 14a,b) to address the use of such SC configurations for outer space missions. In particular, the EQE shows a reduction of the absorption (at wavelength <600 nm, Figure 14a), while the normalized PL map (Figure 14b) reveals the absence of phase transitions and slight variations of *E*_g_ in the investigated temperature range, suggesting the high stability of triple-cation MHPs at these conditions. Interestingly, the authors characterized the PV performance of these PSCs by mimicking the temperature and sun-intensity environment at low Earth orbit, Mars, Jupiter, and Saturn. At low Earth orbit conditions (300 K, AM0), the *J*–*V* curves (Figure 14c) show the typical hysteresis behavior of PSCs (which has been repeatedly assigned to motion of the ions within the perovskite).^158^Figure 14d reports the *J*–*V* curves measured at conditions found on Mars (263 K, 0.43·AM0) characterized by hysteresis behavior and the insurgence of a barrier to current flow for *V* > *V*_OC_. The latter phenomenon is more evident in the *J*–*V* curves measured at Jupiter (135 K, 0.037·AM0) and Saturn (100 K, 0.011·AM0) conditions (Figure 14e,f), while the hysteresis disappears completely (as expected at low temperatures because of “freezed” ion motion).^159−161^ The authors propose that such a parasitic barrier arises at the interface between the perovskite and the electron-transporting layers because of thermionic emission of carriers at the Schottky barrier.^162^ Remarkably, PSCs show good PV characteristics suggesting their possible use in an LILT environment. In fact, the *V*_OC_ and *J*_SC_ reduction are due to only the reduction of the illumination intensity and not to other degradation mechanisms that can affect the stability of the devices.

Finally, at high temperatures, halides (from the MHP) and metal (from the electrode) can diffuse within the charge-carrier extraction layers (especially the organic ones) and form resistive metal halides, causing a drop of the device efficiency (in the case of gold, this process is activated already at ∼70 °C).^144,163^ A promising solution can be the use of carbon-based electrodes which have been repeatedly reported as thermally stable.^164,165^ Moreover, coupling a carbon electrode with CuSCN inorganic HTL recently allowed reaching a PCE exceeding 14.5% with good reproducibility and negligible hysteresis behavior.^166^ As a further step, the PCE gap between gold-based and carbon-based PSCs could be reduced by increasing the conductivity of the carbon paste with the use of graphene fillers, as recently demonstrated by Mariani *et al*.^167^

## Conclusions

Materials used for space applications must withstand severe and hostile conditions, such as the presence of huge quantities of radiation (both directly and indirectly ionizing) and temperature gradients (ranging between −120 and +120 °C for satellites orbiting Earth). Moreover, several practical requirements must be met to reduce production, launching, and maintenance costs as well as increase the resulting performance. In this context, the use of metal halide perovskites (MHPs) for the realization of perovskite solar cells (PSCs) can represent a disruptive solution to the market of space photovoltaics (PVs). In fact, MHPs show great radiation tolerance, rivalling and surpassing that of benchmark materials used for space applications, such as III–V semiconductors making up multijunction solar cells (MJSCs), allowing the reduction of the costs associated with radiation shielding strategies. Furthermore, radiation-tolerant flexible PSCs can also be realized (for example by using polyethylene naphthalate as substrate),^125^ paving the way for the realization of truly rollable solar arrays. However, the mechanisms behind the degradation of PSCs are still the subject of great debate, mainly because it is rather challenging to discern the effects due to illumination and aging from those related to radiation bombardment. It is then clear that further research efforts are needed to shed light on these intricate phenomena. In general, several works point toward a more pronounced degradation of hybrid MHPs (*i.e.*, those with a chemical composition containing organic molecules) with respect to pure inorganic counterparts, suggesting that the use of Cs-based MHPs can be a promising solution for the long-term stability of devices (with current conversion efficiencies exceeding 18% for terrestrial applications).^160^ As such, PSCs are far from being a substitute for the commercially available MJSCs for space applications, but they can offer an important alternative to this technology in those systems where low cost and weight are pivotal. Indeed, the privatization of space exploration asking for smaller and cheaper satellites is revolutionizing the economics of space, providing an ideal niche for the development and commercialization of the perovskite photovoltaic technology. As in the case of terrestrial applications, a promising device structure that can really compete with the III–V-based MJSCs is represented by the tandem architecture. This opens an interesting and challenging research area, because the light harvesters commonly employed as the bottom cell for terrestrial applications are not suitable for use in the space environment. For example, the best-performing Si solar cells are prone to degradation under high radiation doses because of the use of float zone n-type Si wafer with very long carrier lifetime and no specific design consideration for radiation tolerance (as an alternative, the use of a thin Czochralski p-type Si wafer, ∼100 μm, recently started to be tested as a base for producing a thin-Si bottom cell because of its high radiation tolerance).^168^ In the context of radiation-tolerant technologies, two interesting examples of tandem devices are (i) perovskite/Cu(In,Ga)S_2_ (which are radiation resistant, efficient, and lightweight) and (ii) perovskite/perovskite (which were recently recognized as more resistant to proton bombardment with respect to III–V-based MJSCs).^169^ At the same time, the future optimization and testing of PSCSs should take into account the peculiar environment and working conditions experienced by the device during the specific space mission for which they are intended. As an example, mixed-cation-based PSCs were recently investigated under low-intensity/low-temperature (LILT) conditions typical of Mars, Jupiter, and Saturn orbits (missions in deep space).^157^ Indeed, the authors found that the unintentional energy barrier at the FAMACs/SnO_2_ interface, which usually limits the performance of devices under 1 sun AM0 conditions at 300 K, has little effect upon the properties of the devices under LILT conditions. Moreover, looking at the low-temperature device performance, the hysteresis is frozen out and the intensity conditions enable efficient carrier extraction, suggesting that systems showing not excellent performance under standard test conditions could operate well in LILT environments. Furthermore, it is worth mentioning the role that two-dimensional (2D) materials (such as graphene, transition-metal dichalcogenides, and MXenes) can play in enhancing both the performance and the stability of various components (transparent conductive electrodes, charge-transporting layers, and interlayers) of next-generation heterojunction solar cells.^120,170−173^ However, a deep discussion of the role played by 2D materials in PV technologies is beyond the scope of this Review, so the interested reader is encouraged to consult refs (171) and (173−180). In recent years, 2D materials-based devices, such as transistors, sensors, *etc*., have been proposed as future technologies for space applications because of their low weight, small size, and low power requirements. Indeed, several groups demonstrated the radiation resistance of such 2D materials-based systems, paving the way for their use in extra-terrestrial environments.^181−184^ Thus, 2D materials (i) are being used for boosting/improving the performance of PSCs in terrestrial conditions and (ii) show a resistance to radiation that can be exploited for the realization of electronic devices/components used in the space environment. Consequently, we believe that the synergistic use of 2D materials within PSCs can be a promising strategy to produce efficient and reliable next-generation PV technologies.^185^ However, to the best of our knowledge, such devices have not been reported yet, so we encourage the research community to investigate further this aspect. Currently, although PSCs can provide a cheap strategy potentially exploitable for the *in situ* realization of solar arrays during long-term space exploration missions,^42^ there is still significant room for finding the proper materials, architecture, and encapsulation strategies that can really lead to a break-through in the use of such devices for extra-terrestrial (and terrestrial) applications. Finally, prior to the widespread use in the space environment of perovskite technology, the rigorous AIAA-S111 space qualification testing, previously designed for Si and III–V semiconductors, needs to be reconsidered. As an example, for perovskites it may be more appropriate to use lower-energy protons with respect to the current radiation standards, because PSCs have a lower thickness than MJSCs and Si-based devices; thus, high-energy radiation can potentially pass through PSCs without releasing too much energy within the device.^42^ For the same reason, the selection of cell packaging, including the substrate, should be assessed by any ground-based testing and stability validation toward harsher radiation doses. With this in mind, we hope that this work will contribute to stimulate further research efforts regarding this highly interesting and exciting topic.
